# Transgenic tomato strategies targeting whitefly eggs from apoplastic or ovary-directed proteins

**DOI:** 10.1186/s12870-024-05852-5

**Published:** 2024-12-27

**Authors:** Natalie S. Thompson, Aliya Fathima Anwar, David Krum, Michael Ream, Eva Shouse, Zachary Weston, Yun-Ru Chen, Aisa Sam, Michihito Deguchi, Samwel M. Kariuki, Sairam V. Rudrabhatla, Wayne R. Curtis

**Affiliations:** 1 Department of Chemical Engineering, The Pennsylvania State University, University Park, PA 16802 USA; 2 Microbiology Program, The Pennsylvania State University, University Park, PA 16802 USA; 3 Biotechnology Program, The Pennsylvania State University, University Park, PA 16802 USA; 4 Department of Biology, The Pennsylvania State University, Harrisburg, PA 17057 USA; 5 Intercollege Program in Plant Biology, The Pennsylvania State University, University Park, PA 16802 USA; 6 African Genome Center – UM6P, Ben Guerir, Morocco; 7 Department of Plant Sciences, Kenyatta University, Nairobi, PA 16802 Kenya; 8 Department of Genetics, University of Georgia, Athens, GA 30602 USA

**Keywords:** Whitefly, Egg sterilization, Insecticide, Tomato, GM crop

## Abstract

**Background:**

Transgenic plants expressing proteins that target the eggs of the ubiquitous plant pest Bemisia tabaci (whitefly) could be an effective insecticide strategy. Two approaches for protein delivery are assessed using the mCherry reporter gene in transgenic tomato plants, while accommodating autofluorescence in both the plant, phloem-feeding whitefly and pedicle-attached eggs.

**Results:**

Both transgenic strategies were segregated to homozygous genotype using digital PCR. The first strategy uses a glycotransferase secretion signal peptide. Despite bright apoplastic accumulation, mCherry is not evident in the eggs. The second strategy targets in vivo whitefly eggs, where the mCherry transgene was fused to a protein transduction domain (PTD) to facilitate uptake into the whitefly hemolymph as well as a synthetic vitellogenin ovary-targeting sequence. Phloem-specific expression of the mCherry fusion is achieved from a Commelina viral promoter. Accumulation was not sufficient to be observed in females feeding on these ovary-targeting plants nor in their eggs subsequently laid on non-transgenic plants. Egg protection may be mediated by protease activity which is observed in macerated eggs.

**Conclusions:**

mCherry proved an effective reporter for the desired tissue-specific expression in tomato, but insufficiently sensitive to allow for localization in feeding whiteflies or their eggs. Segregated homozygous transgenic tomato lines were important for drawing these conclusions. The implications of these observations to possible pest-control strategies including preliminary expression of analogous chitinase constructs are discussed.

**Supplementary Information:**

The online version contains supplementary material available at 10.1186/s12870-024-05852-5.

## Background

Insect herbivory generally results in obvious and sometimes dramatic plant damage due to the large volume of biomass consumed. Most of the damage done by whiteflies, in contrast, is not biomass consumption but a result of their phloem-feeding behavior. Whiteflies participate in both non-persistent and life-long persistent virus transmission as well as promotion of microbial growth due to their ‘honeydew’ excretion caused by stoichiometric excess of phloem sugar relative to nitrogen [1, 2]. Consistent with the reported broad host range of several hundred species for whitefly [3], we have demonstrated the successful *Bemisia tabaci* MEAM1 proliferation on several dozen different plant species [4]. Throughout this broad host range, the relationship between whiteflies of the genus *Bemisia* and their host plants has been shown to be quite complex; whitefly survival and host plant selection have been correlated to factors affecting the plant-leaf microenvironment including trichome density, leaf nitrogen [5] and sucrose content [6]. Specificity between whiteflies and their host has been shown to develop, as the growth rate of Hungarian whiteflies on Hungarian sweet pepper cultivars has been shown to be greater than that of Hungarian whiteflies reared on Dutch sweet pepper cultivars, and vice versa [7]. As the natural vector for begomoviruses, whiteflies contribute to significant crop losses from the tomato leaf curl virus [8], the lettuce infectious yellow virus [9], the bean golden mosaic virus, and the African cassava mosaic virus [10]. Annual losses in cassava as the major food crop of Africa are estimated to be valued at $2 billion [10] and contribute to widespread hunger and food insecurity.

**Fig. 1:**
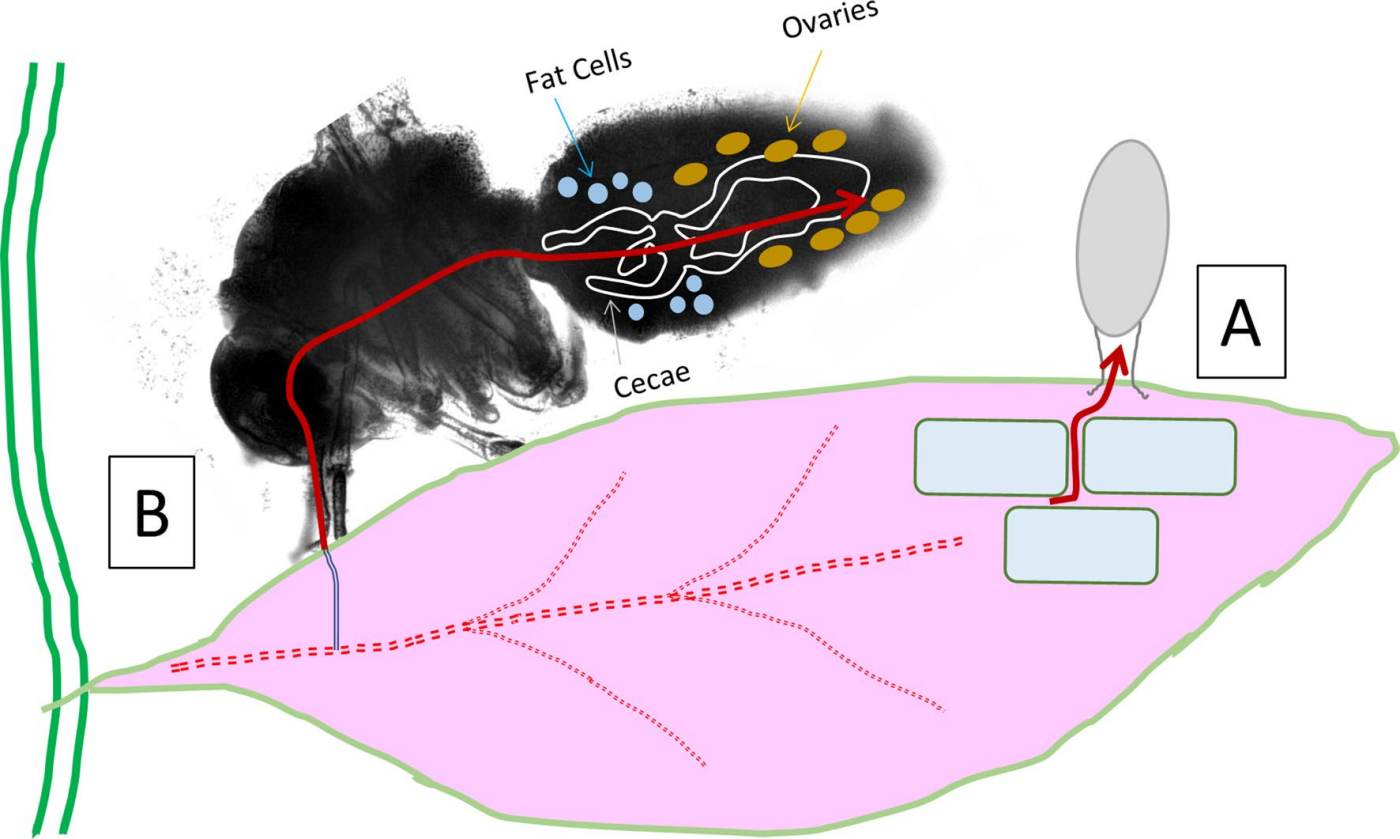
Alternative routes of plant proteins into whitefly egg. (**A**) Egg pedicel is imbedded into apoplast and known to facilitate uptake of as much as 50% of egg water volume. (**B**) Whitefly feeding from phloem combined with protein transduction domain into hemolymph and ovary-targeting vitellogenin motif fusion for targeted egg protein loading

Conversely, viruses can be viewed as providing ecosystem stability by preventing overgrowth of homogeneous populations and even promoting adaptation to changing environments [11]. The focus on plant disease has fundamentally overlooked the potentially beneficial roles of plant viruses [12]. Considering the efficiency with which phloem-feeding insects can deliver viral DNA, we have been exploring their potential to be harnessed for plant gene therapy. Responsible implementation of such a technology has also motivated our study of whitefly pest management. In this context, there would be value to developing GM plants that could attenuate whitefly proliferation by targeting eggs rather than the adult insect vector. This strategy has fundamental differences from the typical insecticidal approach that target the adult such as toxins [13] or silencing RNA [14]. These reviews cover the dozens of permutations from transgenic, viral vectors, symbionts that range considerably in their specificity, effectiveness and cost, where the targeting fecundity while maintaining healthy adults is rarely mentioned [13]. The deposition of insect eggs on plants has been shown to elicit plant defense response [15, 16], which can provide opportunities for future implementation in the specificity of GM strategies. An advantage of our proposed approach to target eggs has the advantages of biological specificity, while also being compatible with proven insect control strategies such as the sterile insect technique [17, 18]. Figure 1 reflects two strategies to target whitefly eggs with GM host plants. The leaf apoplastic path is based on flux of leaf nutrients into the whitefly egg which is simpler and rationalized below first. The alternative phloem-feeding insect path is based on the signals that traffic egg storage proteins (in females) from the site of synthesis in the insect fat body, through the hemolymph, and into the developing oocytes.

The pedicel of the whitefly egg, which connects the egg to the leaf tissue, has been shown to transport substantial water (as much as half its volume) into the egg from the host plant and is critical to the survival of the egg to hatching. Osmotic pressure drives water diffusion across the cell wall, into the apoplastic space, and then into the insect egg through a layer of an adhesive colleterial gland secretion [19]. Direct insertion of the pedicel fibrils into abaxial epidermal cells has also been observed, suggesting that cytoplasmic solutes may also enter the whitefly egg through the pedicel [6]. Moderate molecular weight carbohydrate polymer ([14 C]-Inulin) has been shown to be transported into the egg, proving that solutes of considerable size can be taken up by the whitefly eggs. These observations suggest that there may be an opportunity to deliver inhibitory proteins from a transgenic plant to the egg for a transgene expressed into the apoplastic space. Secretion of proteins out of a plant cell into the apoplast is mediated by N-terminal export signal peptides. These signal peptides are typically less than 100 amino acids which are cleaved from the protein as it is directed for secretion into the apoplastic space. The associated signal cleavage site can be predicted with increasing accuracy [20, 21] such that the peptides can be fused to plant transgenes to facilitate their export into the plant apoplastic space. Our choice of signal peptide was based on proteins with apoplastic functions based on UniProt descriptions and influenced by some prior work including homologs of phytocyanin-like arabinogalactan (PLA) [22]. The rationale for prioritization in pursuing transgenics with successfully cloned export signal peptides using the program SignalP is described in Supplemental File A.

Alternatively, the potential ovary-targeting route of transgenic proteins to the whitefly egg is to ‘hitchhike’ on the pathway for egg storage proteins (Fig. 1). The dominant egg storage protein vitellogenin (Vg) is predominantly synthesized in the insect fat body [23] as one of the most highly expressed whitefly transcripts [24]. Upon traversing the insect hemolymph, a targeting sequence facilitates specific uptake of Vg into the developing oocyte. This could be accomplished from whitefly feeding on a plant expressing an insecticidal transgene protein that includes a protein domain to facilitate transport from the gut into the hemolymph. Therefore, the proposed ovary-targeting approach to delivering a transgenic plant protein must contain both the cell penetrating protein transduction domain (PTD) as well as a synthetic vitellogenin signaling polypeptide (SynVg). The options of a protein transduction domain are limited. Tat and VP22 are both PTDs derived from human viruses (HIV and HSV respectively) where penetratin is a domain of the Antennapedia protein of fruit fly (*Drosophila melanogaster*). The insect origin of penetratin and its lack of a requirement for receptor mediated endocytosis [25] made this a logical candidate for this study. Our design of the synthetic vitellogenin domain (SynVg) involved a bioinformatic comparison of GenBank Vg sequences of multiple strains of whitefly as well as additional bioinformatic comparisons to the literature of Vg sequences (see Methods and Supplemental File B).

## Methods

### Tomato plants

The *Lycopersicon esculentum* variety utilized was ‘Florida Lanai’, was a gift from Dr. Jane Polston, University of Florida. This miniature tomato was originally developed as an ornamental [26] but provides for convenient small-scale proliferation in growth cages. Tomato plants were grown on lighted racks inside insect cages (either 12”W X 24”L X 18”H with a 12” x 12” front panel door with a reach-in sock and upper vinyl view panel BioQUIP #1450NS85 or 24” cube BioQUIP #1450NS78).

### Whitefly colony

Whitefly (*Bemisia tabaci* Biotype B, Middle East Asia Minor 1, MEAM1) was maintained by serial transfer on cabbage (*Brassica oleracea*, var. Earliana, W. Atlee Burpee Company) as a simplified qualitative version as described previously [27]. Cabbage maintenance was in part a biocontainment strategy for USDA permitted work on genetically engineered begomoviruses – for which cabbage is not a host. Whiteflies were also maintained in a larger format 24” cubical screen cage (BioQUIP #1450NS78) with two cabbage plants initiated from five week old seedlings in a 6-in DIA pot and permitted to grow for a month before the addition of 25–40 whiteflies. This procedure allowed for long-term, low effort maintenance of bi-monthly subculture that can provide whiteflies for several months as needed.

### Whitefly proliferation model

We have previously published a quantitative whitefly proliferation model that was parameterized based on our observations of prolific growth on cabbage. Since whiteflies are known to have different fecundity on different hosts [28], we carried out a comparable assessment of whitefly proliferation on five ‘Florida Lanai’ tomato plants in the 12 × 18 × 24 inch cage format noted above to assess the innate insecticidal nature of tomato. Whitefly accumulation on tomato was observed to be about 20% for comparable conditions with cabbage (see Supplemental File E). Additionally, we performed single plant fecundity studies in small 12 × 12 × 12 inch cages with a front vinyl window.

### mCherry reporter gene choice

Autofluorescence is known to be problematic for the use of fluorescent reporters for both plants [29] and insects [30]. Choosing a fluorescent protein that is compatible for the combined study of tomato and whitefly is challenging. The broad range of autofluorescent compounds in plants spans the entire visible spectrum [31] with considerable broad autofluorescence in lower visible wavelengths (e.g. lignin ~ 460 nm) as well as prominent photosynthetic pigments (e.g. chlorophyll ~ 680 nm). Preliminary screening of whiteflies and whitefly eggs for autofluorescence by scanning excitation up to the dichroic mirror cutoff indicated strong whitefly autofluorescence for excitation in the 500 nm range (see Supplemental File C, Figure SC10 & SC11).

**Fig. 2:**
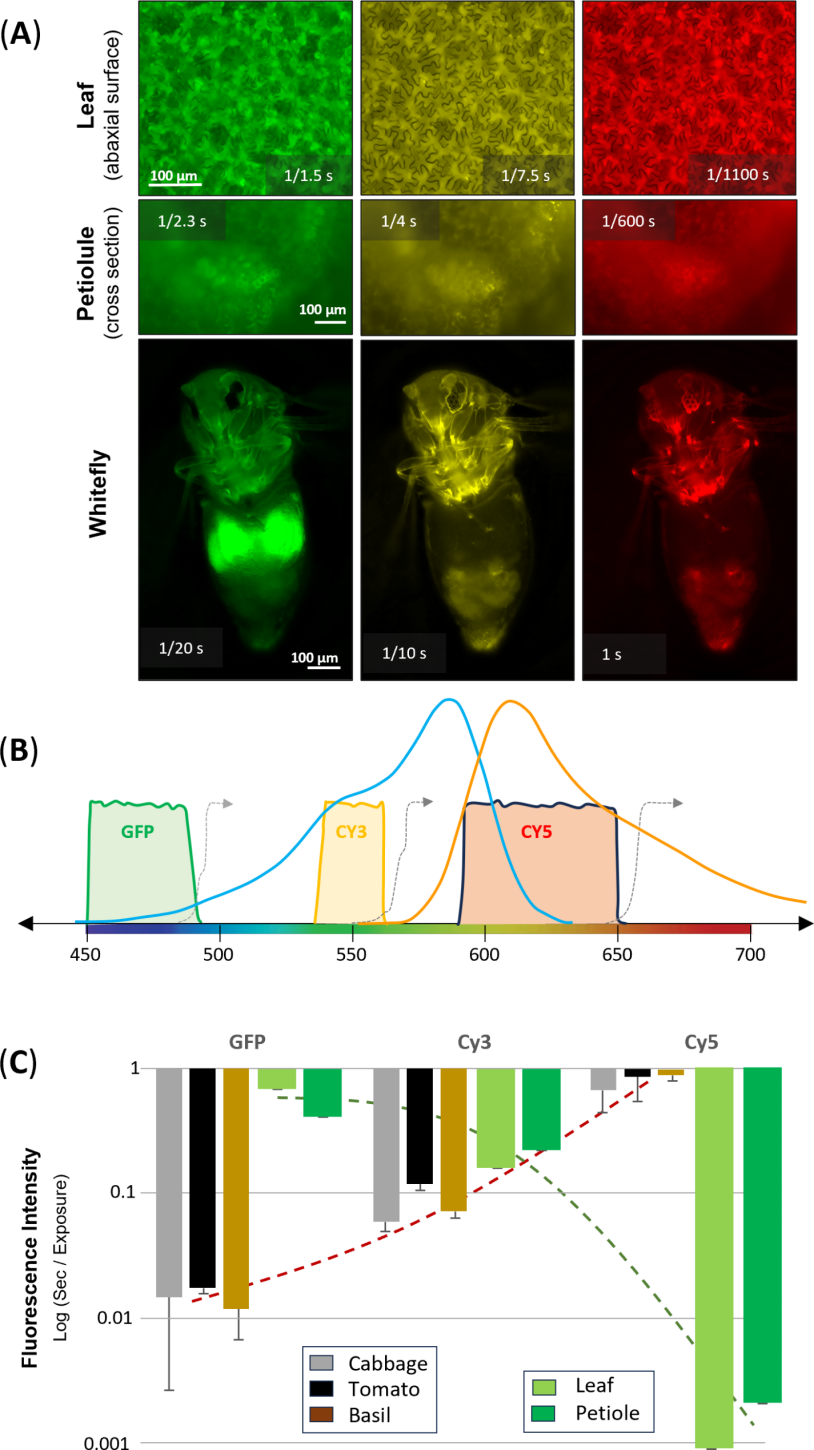
Assessment of autofluorescence in tomato and whitefly as basis of choosing fluorescent protein reporter. (**A**) Confocal microscopy images taken at the exposure time of each image that produced ‘incipient saturation’ for wild type tomato and whitefly for the three different fluorescent filter sets. Exposure times are shown in each image. (**B**) Optical characteristics for fluorescent excitation / imaging using the GFP, Cy3 and Cy5 filter sets of the confocal microscope. The grey dashed line represents the dichroic mirror cut-off. The excitation (blue) and emission (orange) for mCherry florescent protein is provided on the same visible spectrum scale. (**C**) Autofluorescence graph based on ‘incipient saturation’ to quantify autofluorescence (larger bar = more autofluorescence) to compare the tradeoff of competing autofluorescence for whitefly (gray, black, brown after feeding on three different plant hosts) and tomato (green bars)

The operational characteristics of the KEYENCE confocal microscope used for most fluorescence observations provided a generalized approach to translating these visual observations to quantitative measurements of fluorescence (Fig. 2). A reproducible quantitative measure of fluorescence intensity was obtained by incrementing the exposure time to the point at which the digital image would saturate the imaging screen brightness; the exposure time that provided a non-saturated exposure was termed ‘incipient saturation’. This also allowed for using the confocal image to focus on localized expression. The images in Fig. 2A represent ‘incipient saturation’ for tomato (abaxial leaf surface, as well as petiolule cross sections) and whitefly. These representative images are aligned with the confocal microscope ‘excitation window’ and paired dichroic mirror for three fluorescence filter cube sets (Fig. 2B). Superimposed on this visible spectrum, is the excitation and emission profile of mCherry. Noting that exposure times were less than 1 s, a log (base 10) plot of the exposure time becomes an indicator of the level of the background interfering autofluorescence (Fig. 2C). In this manner, a method was established to quantify a basis for choosing a fluorescent protein that could avoid the high autofluorescence of insects at low wavelengths such as typical use of GFP in transgenic plants, while also avoiding the high autofluorescence of plant pigments in the higher wavelengths. Within this range, mCherry was chosen as the compromise fluorescent protein reporter. Noting that the mCherry fluorescent protein characteristics (excitation max 587 nm, emission max 610 nm) is a misnomer with regard to the visible spectrum (yellow 565–590 nm, orange 590–625 nm) we have chosen to pseudo-color yellow for mCherry – as this also provides for higher contrast in visualization. The version of mCherry utilized corresponds to a minor variation of the native sequence with the N-terminal truncation to eliminate the alternative translation mCherry isoform [32]. The sequence can be found in the GenBank submissions of the transformation constructs noted below.

### Apoplastic transformation binary vector design

The apoplastic transformation vector was based on the pEAQHT binary vector previously utilized in our laboratory [33] derived from the vector kindly provided by George Lomonossoff [34, 35]. Not all constructs were successfully progressed to final transgenic plants but are described here and will be made available (GenBank, ADDGENE: www.addgene.org/Wayne_Curtis/).

### Signal peptide choice

Several tomato genes were chosen to identify homologous tomato secretion signal peptides; this included early nodulin-like protein 3 (XP_004244524) which is a known extracellular embryogenic arabinogalactan protein (LePLA1). This yielded a 25-aa signal peptide [MAAKAFSRSITPLVLLFIFLSFAQG] with favorable Y-score (combined cleavage site score) in SignalP4.0 [20] (Y = 0.868). Similarly, a glycotransferase involved in primary wall biosynthesis, xyloglucan endotransglucosylase/ hydrolase 1 (LeXHT1 = Q40144 by similarity) provided a 22-aa signal peptide [MGIIKGVLFSIVLINLSLVVFC] with Y = 0.497. These signal peptide amino acid sequences were synthesized as g-block fragments from IDT (idtdna.com).

### Apoplast targeting T-DNA assembly

The signal peptides were appended to the N-terminal of mCherry using overlap extension PCR. Each signal peptide was PCR amplified with extension or restriction primers along with the corresponding primers for mCherry: LePLA1 signal peptide (Fwd- 5’-tcgcg**accggt**ATGGCTG-3’ [AgeI bold], Rev-5’-GCTTCACAGAAGCAatggccatcatc-3’ with mCherry Fwd- 5’ gcttcacagaagcaATGGCCATCATCAAGGAG-3’and Rev- 5’ cactctcgagTTACTCGTCCATGC 3’) and LeXHT1 signal peptide (Fwd- 5’- ttcgcgaccggtATGGGTATC, Rev-5’- gatgatggccatCCCACAAAATACAACAAGTGAC 3’ with mCherry Fwd-5’-CGTATTTTGTGGGATGGCCATCATCAAGGAGTTC-3’and Rev-5’ CACT**CTCGAG**TTACTCGTCCATGC 3’ [XhoI bold]). Fusion of signal: mCherry was accomplished by running these fragments with adjacent primers for 15 cycles, then the restriction site overhang primers from each end were added to produce the fused product which was restricted and ligated into the pEAQHT vector linearized with AgeI and XhoI. This cloning strategy retained the vector P19 anti-gene silencing element as well as the 5′-untranslated region (UTR) and the 3′‐UTR from CPMV RNA‐2 is that of the original pEAQ vector. These binary vectors, designated Ly60, pEAQHT//35s: P19::sigLeXH1:mCherry: NosT, and Ly66, pEAQHT//35s: P19::sigLePLA1:mCherry: NosT were confirmed by restriction map and full plasmid sequencing (plasmidsaurus.com) for submission to GenBank: accession OR636129 and OR695066 respectively.

### Ovary targeted transformation binary vector design

The ovary-targeted design included consideration for vascular-system specific expression at the site of whitefly phloem feeding as well as the protein transduction and synthetic vitellogenin domains to facilitate transport to the ovaries of a feeding female whitefly. The mCherry construct was generated in two orders: PTD-SynVg-mCherry and PTD-mCherry-SynVg. The former is the basis of the transgenics described in this work, where the latter was also generated, and regenerated tomato plants were selfed for T1 seed, but lost due to improper seed harvest during constraints of COVID research activities.

### Protein transduction domain

The protein transduction domain of *Drosophila*, amino acid sequence: RQIKIWFQNRRMKWKK, was synthesized by TWIST Bioscience (twistbioscience.com) based on codon optimization for tomato to yield (AGGCAAATCAAGATTTGGTTTCAAAACCGAAGGATGAAGTGGAAAAAA).

### Vitellogenin ovary-targeting domain

**Fig. 3:**
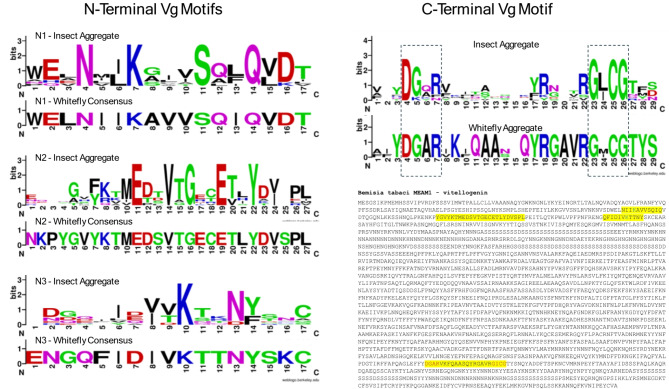
Sequence Logo alignment of insect vitellogenin (Vg) protein domains identified as interacting with the receptor for egg uptake. Insect aggregates include Whitefly (*Besimia tabaci*), planthopper (*Laodelphax striatella*), mosquito (*Aedes aegypti*), Asian ladybug (*Harmonia axyridis*), silkworm (*Bombyx mori*), palm beetle (*Octodonta nipae*), tomato bug (*Nesidiocoris tenuis*), parasitic wasp (*Pimpla nipponica*), and Tibet moth (*Thitarodes pui*). Whitefly aggregate sequences include MEAM1, Asia-I, ZHJ-II and Biotype Q where N-terminal sequences were identical. The MEAM1 Vg sequence illustrates the distance between the N- and C- terminal sequences which required a condensed synthetic vitellogenin (SynVg) fusion tag synthesized based on tomato codon optimization. See Supplemental File B for more details

The design of the synthetic vitellogenin domain (SynVg) involved an extensive bioinformatic analysis that is presented in Supplemental File B. Figure 3 presents the key consensus Vg-receptor (VgR) binding motifs that were considered in the SynVg design based on amino acid consensus sequence alignments and Vg-VgR interaction studies.

The DGxR—GL/IGC C-terminal motifs have been described in numerous characterizations of insect vitellogenins [36, 37]. A prawn (*Macrobrachium rosenbergii*) peptide array pull-down and mass spectrometry study focused attention on the N-terminal binding domains [38] for alignment against the whitefly Vg sequence. This analysis resulted in Vg motifs associated with interaction with the Vg receptor (VgR) for uptake by the egg that are spaced over 1000 amino acids apart. Based on prioritizing the studies which included physical binding, only the N-terminal sequences were used. This bioinformatic analysis converged on a 54-aa sequence WELNIIKAVVSQIQQNLKKSSYKTMEDSVTGECETLYDVSQFIDIVKTTNYSKC. The tomato codon-optimized sequence as reflected in the GenBank accessions, was synthesized by TWIST to generate the SynVg targeting element.

### Ovary targeting mCherry and chitinase T-DNA assembly

The phloem specific Commelina yellow mottle virus promoter (pCoYMV) kindly provided by Professor Neil Olszewski was amplified and ligated into the pLSU2 binary vector using CoYMV SaII Fwd 5’ TTTA**GTCGAC**ATCGATTTCTTAGGGGCTTCTCTCGG 3’ and CoYMV SacII Rev 5’ CCC**CCGCGG**GGATCCTTGTTGTGTTGGTTTTCTAAG 3’ with restriction sites bolded. The terminator NosT was PCR amplified and cloned using primers NosT MluI Fwd 5’ TTTT**ACGCGT**GATCGTTCAAACATTTGGCAAT 3’ and NosT XhoI Rev 5’ CCC**CTCGAG**GATCTAGTAACATAGATGACACCG 3’. The protein transduction domain (PTD) and synthetic vitellogenin domain (SynVG) and mCherry were then assembled in two orders PTD: SynVG: mCherry and SynVG: mCherry: PTD, placing the protein transduction domain in the N-terminal and C-terminal respectively. The individual fragments were PCR amplified with overlap primers and the purified products were mixed and annealed for 15 cycles without primers. The end primers with restriction sites were added and PCR cycled for an additional 20 cycles. For cloning in the pLSU2 binary vector, the correct size fused fragments were gel extracted and subsequently ligated into the vector. Additional details of codon-optimized gene sequences and fusion PCR primers are described in Supplemental File G. The binary vectors, designated Ly62 = pLSU2//pCoyMV: PTD: SynVg: mCherry: NosT, and companion vector, designated Ly65 = pLSU2//pCoyMV: SynVg: mCherry: PTD: NosT were confirmed by restriction map and full plasmid sequencing (plasmidsaurus.com) for submission to GenBank: accession OR695065 and OR695069.

Additional binary vectors were created using the insecticidal fern chitinase Tma12 [39] that was tomato codon-optimized and synthesized using TWIST and inserted as the transgene replacement of mCherry. Cloning was analogous to the process as above, but with respective overlap primers binding to the chitinase gene. Additional codon-optimized gene sequences and fusion PCR primers are described in Supplemental File G. The designations for these insecticidal constructs are: Ly61 = pLSU2//pCoyMV: PTD: SynVg: Chitinase: NosT, GenBank accession OR695068, and Ly64 = pLSU2// pCoyMV: SynVg: Chitinase: PTD: NosT, GenBank accession OR695067. Additional cloning details, sequences, primers, and restriction enzymes are described in Supplemental File G.

### Tomato transformation and seed production

The Agrobacterium tomato cotyledon transformation protocol was adopted (with slight modifications) from Arshad et al., 2014 [40] using the Cys-32 Agrobacterium auxotroph developed in our lab [41]. Tomato seeds of *Lycopersicon esculentum* (FLA ‘Lanai’) were surface sterilized with 5% v/v commercial bleach (6% w/v Na-hypochlorite) with 2–3 drops of Tween-20 surfactant per 100 mL and germinated on solidified hormone-free ½ strength MS salts media [42]. Agrobacterium was grown on selective media (50 mg/L kanamycin, 20 mg/L rifampicin) for ~ 2 days and resuspended in ½ MS salts with 200 µM acetosyringone for vir gene activation. Cotyledons and hypocotyls were excised at ~ 9 days; after suspension of the cotyledon explants for 20 min for gentle shaking, blotting, and cultivated in the dark for 48 h, adaxial side down on sterile filter paper and placement on filter paper on MS salts agar medium. Explants were then washed with ½ MS salts liquid medium containing 500 mg/L cefotaxime and plated on 100 mg/L kanamycin selection media (MS salts agar with 500 mg/L cefotaxime, 150 mg/L timentin, 2 mg/L zeatin, and 0.1 mg/L IAA). Explants were then transferred bi-weekly to fresh selection media to prevent Agrobacterium overgrowth and observe plantlet regeneration. Independent transformants (based on individual explants) emerged as shoot primordia were transferred to shoot induction media with reduced zeatin: 0.1 mg/L zeatin, and 0.1 mg/L IAA (with continued antibiotics for Agrobacterium; 500 mg/L cefotaxime, 150 mg/L timentin, and selection 100 mg/L kanamycin). Once regenerated plants have discernable shoot, they were moved to root induction media with further reduced plant hormones to 0.05 mg/L indole-3-butyric acid (IBA) and continued 500 mg/L cefotaxime, and transgene selection 100 mg/L kanamycin. Rooted transgenics were then transferred to soil and grown for several weeks (while being tested for transgene for PCR, including a primer set for the Agrobacterium nptI promoter (Fwd = CCACGTTGTGTCTCAAAATCTC, Rev = AACACCCCTTGTATTACTGTTTATG) as control to assure absence of the Agrobacterium transformation vector. Potted plants were grown to seed in a greenhouse, self-pollinated T1, T2 and subsequent transgenic segregations.

Tomato seeds were harvested by cutting fruit into quarters and pressing out the seeds. The resulting tomato seed puree was fermented overnight to facilitate breakdown of the tomato tissue, followed by a 5-minute exposure to ~ 5% by volume bleach solution and rinsing under cold water for at least 15 min. After drying seeds at room temperature, the seeds were then stored at 5 °C.

### Imaging

Imaging was conducted on numerous microscope platforms. Routine screening was conducted on a custom-designed optical bench with the Thorlabs (thorlabs.com) epifluorescent microscope CEA1400 microscope in a light-proof enclosure fabricated from T-slot hardware and 1/8” PVC black plastic sheet (US plastics, cat #45093) on top of a ThorLab optical vibration table. Image capture was with an 8.0 MP CCD camera (8051 M-USB-TE) and a 10X Nikon Plan Fluorite objective (N10X-PE), provided for full view of the whitefly and eggs. The mCherry fluorescence excitation was with a 565 nm LED (M565L3) with a filter set from Semrock: 593 nm dichroic (FF593-Di03-25 × 36), 575 ± 15 nm (FF01-575/15–25), and 641 ± 75 nm (FF02-641/75 − 25) bandpass filters. The acquired images were colorized in ImageJ (Fiji) (64-bit Java 1.8.0_172).

Additional imaging was conducted utilizing a KEYENCE BZ-X Confocal Microscope platform using a x10 Plano Apochromatic objective (BZ-PA 10) and Chroma EY Cy3/TRITC filter cube (excitation 520–570 nm; emission 570–640 nm) for mCherry (excitation max 587 nm, emission max − 610 nm). Leaf petiole tips (third/fourth true leaf) at the fifth/six leaf seedling stage were imaged from abaxial surface under a cover slip. Phloem-targeted expression was assessed using free-hand scalpel sections of the petiolule from young tomato seedlings. Petiole sections could be quickly visualized without a cover slip on the inverted KEYENCE slide stage. Free-hand sections of roots, stems, and flower parts (anther, ovary, stigma, petals, and sepal/calyx) were imaged under cover slips; notably anthers were otherwise prone to rapid oxidation that would produce artifacts of autofluorescence in the Cy3/mCherry imaging window. Whiteflies were imaged by careful placement on a thin film of microscopy emersion oil after cold-stunning the whiteflies. Whitefly eggs / pedicels were imaged after gentle brushing of eggs off leaves using a cold 10 g/L NaCl solution, followed by 2-minute exposure to 5% bleach to reduce surface protein contamination. To provide more specific excitation, additional confirming imaging utilized a variable wavelength laser excitation (Olympus Fluoview FV10i, FV10C-HOS-2 slide stage).

### Fluorescence analysis of plant and whitefly extracts

Protein extracts and phloem exudates were analyzed on TECAN M Plex 200 fluorometer at an excitation of 585 nm and emission sensing at 620 nm. Leaf protein extracts were obtained by collecting a 100 mg sample and dipping in liquid nitrogen before adding protein extraction buffer at a 4 mL/mg leaf tissue ratio. Whitefly protein extracts after 2.5 days of feeding were obtained from 10 eggs or 10 whiteflies per 250 µL protein extraction buffer; samples were ground with a sterilized plastic epitube pestle, centrifuged, and the supernatant was collected and used directly for fluorescence scanning. Phloem exudates were collected as previously reported for our proteomic studies [43]. Briefly, phloem exudate of transgenic and wild-type tomato plants was collected from leaflets excised at the petiolule using a scalpel. The excised leaf was transferred immediately into a solution containing 20 mM EDTA for 30 min in a humid chamber followed by immersion in 250 µL distilled water in an epitube to collect exudates for six hours in the dark.

### Digital PCR transgene segregation

Digital PCR was performed by The Penn State Genomics Core Facility at University Park using the QuantStudio 3D Digital PCR System according to the manufacturer’s protocol (ThermoFisher). Digital PCR was used to assess gene copy number [44] where PROSYSTEMIN (SYS) is used for the reference gene based on primers (Fwd = GCAATATCAAGAGCCCCGTC, Rev = ATGTGTGCTAAGCGCTCC) to produce a 91-bp amplicon. Transgene insertion copy number was based on the linked kanamycin selectable marker (nptII, Fwd = TTGCCGAATATCATGGTGGA, Rev = TCAGCAATATCACGGGTAGC) to produce a 113-bp amplicon. Digital PCR operated at 60 ^o^C using HEX™-labeled probe for nptII (5’HEX/CCGGCCACA/ZEN/GTCGATGAATCC/3’IABkFQ double-quenched with ZEN and Iowa Black Hole Quencher) and FAM™-labeled probes for SISYS (5’6-FAM/TGCAACATC/ZEN/CTTCTTTCTTCTCGTG/3’IABkFQ). Leaf samples for DNA extraction were typically harvested at the 4-leaf stage. Leaf tissue is frozen in liquid nitrogen before being ground in a BioSpec mini-beadbeater. DNA is then extracted using the MasterPure™ Complete DNA and RNA Purification kit according to manufacturer instructions to provide a minimum required yield of 300 ng/µL (typical 1,460 ng/µL was obtained). DNA concentrations were determined by Qubit dsDNA assay and then diluted to 14 ng/µl (based on genome size). Probe (8 µL) and primer (18 µL) at 4 µM were combined with the reaction mix: 7.25 µL dPCR master mix, 2.9 µL primer/probe mix, and 3.85 µL DNA sample.

## Results

### Vector construction and functionality

**Fig. 4:**
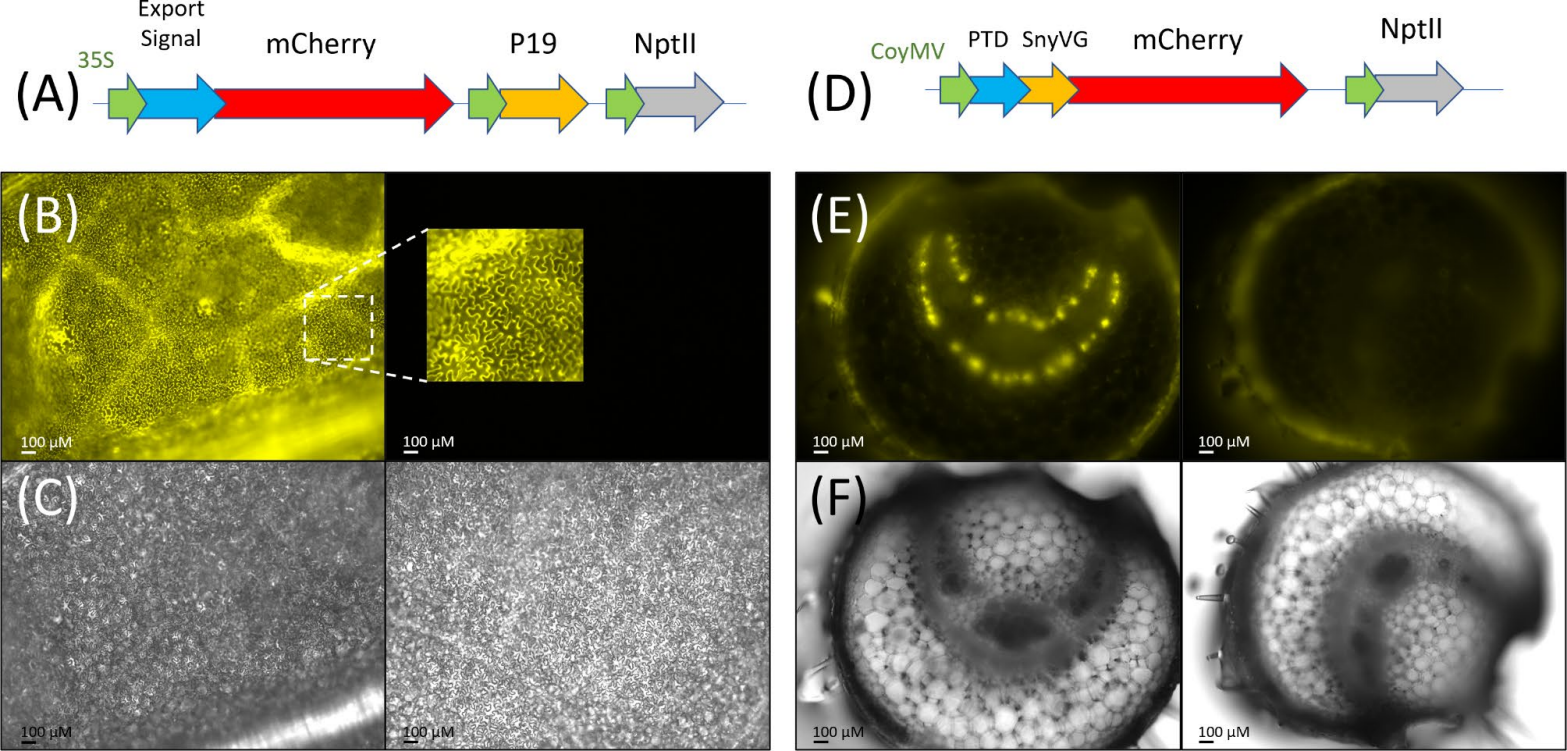
T-DNA constructs and confocal images of transgenic plants. (**A**) Ly60 mCherry with an apoplastic export signal peptide and P19 anti-gene silencing. (**B**) Transgenic Ly60 tomato confocal image (1/300 s exposure) with expanded area overlay on adjacent wild-type tomato confocal image at same settings. (**C**) Brightfield images of the same areas as B. (**D**) Ly62 mCherry fusion with gut protein transduction domain (PTD) and synthetic vitellogenin ovary targeting (SynVg) driven by Commelina virus phloem specific promoter (pCoyMV). (**E**) Petiole cross-section of transgenic Ly62 tomato confocal image (1/20 s exposure) adjacent to a wild-type segregant. (**F**) brightfield images of same areas as E. Fluorescent images with 550–570 nm excitation, 570–640 nm emission; brightfield images identical field of view

Apoplastic and vascular-specific expression of mCherry shown in Fig. 4A and D respectively, represent the successful outcome of a five-year effort to generate transgenic plants designed to assess the feasibility of targeting insecticidal gene expression towards egg viability rather than the adult insects.

The implementation of the milestone-driven DARPA project (https://www.darpa.mil/program/insect-allies) involved starting with a breadth of options which would undergo down-selection based on a combination of timeline, progress and resource prioritization that is considerably different from typical academic research. This is particularly relevant in the context of GM plant development due to the protracted timelines which require decisions to be made years in advance of testing. As a result, extensive resources were created but not followed up – though available for future research.

The mCherry reporter gene truncated to avoid interfering mCherry isoforms provides exceptional signal-to-noise visualization in transgenic tomato and is apparently minimally affected by the omission of the three C-terminal amino acids necessitated by PCR primer design. The LeXHT apoplastic export signal combined with the P19 antigene silencing (Ly60) resulted in highly expressed mCherry that is particularly bright in the apoplastic spaces of the plant leaf (Fig. 4B and Supplemental File C, Figures SC1, SC2). The analogous apoplastic construct based on the LePLA1 signal peptide (Ly66) was also constructed but did not produce transgenics in the initial transformation. As illustrated in the petiolule cross section, the Commelina yellow mottle virus promoter (pCoYMV) provides a distinct phloem-localized expression of mCherry (Fig. 4E). A permutation of the ovary-targeted construct of particular interest was to locate the protein transduction domain (PTD) at the C-terminal [SynVg::mCherry::PTD] to provide spatial separation from the synthetic vitellogenin targeting sequence (SynVg). This (Ly65) construct was created and confirmed as well as the generation of multiple T_0_ transgenics, however, viable seed was unfortunately not successfully collected due to seed harvest mishandling during COVID- pandemic research constraints. The analogous construct to Fig. 4D replacing mCherry reporter with the Tma12 chitinase (Ly64) as well as the C-terminal PTD version (Ly65) were also generated and used to create transgenic tomato lines that will be briefly discussed.

### Apoplastic transgenic characterization

**Fig. 5:**
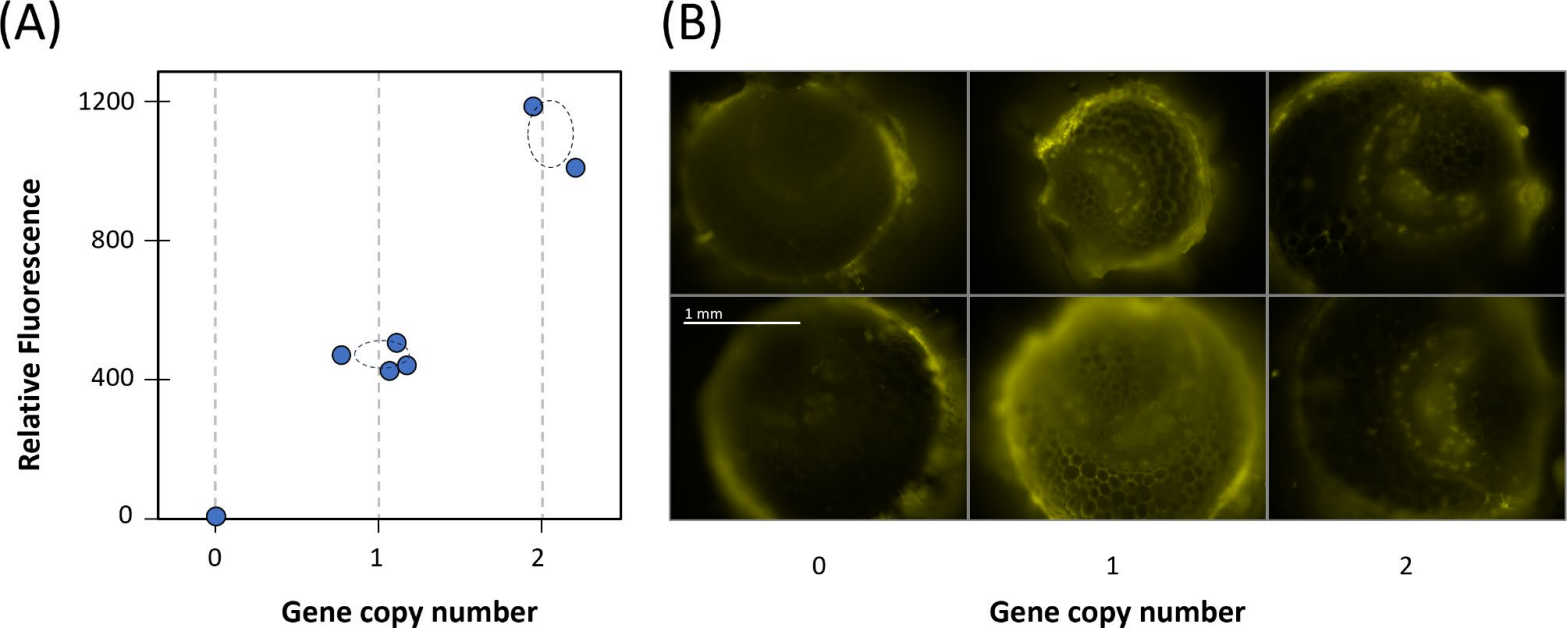
Segregation of hemizygous transgenics for the apoplastically expressed (Ly60) and phloem-targeted (Ly62) expression of mCherry. (**A**) Protein extract fluorescence as basis of confirming mCherry expression and transgene segregation from T_2_ segregants from a T_1_ hemizygous single gene copy insertion for Ly60; fluorescence (ex. 585 nm, em. 620 nm; 96-well plate) plotted against dPCR gene copy number with confidence interval (STD) dashed ovals and (**B**) Images of Ly62 with confocal microscopy (1/20 s exposures, Cy3 filter set) aligned by gene copy number. All images taken at the same magnification

The initial *Agrobacterium* transformation of roughly 100 tomato cotyledon explants with the Ly60 apoplastic construct produced two regenerated transgenics. Both tested PCR positive for mCherry (and negative for *Agrobacterium* genes). Both also displayed relatively intense apoplastic expression with simple and confocal fluorescence microscopy (Fig. 4B). The Ly60.1 transgenic survived as a sucker propagule for over a year, during which time the development of digital PCR provided for an assessment of gene copy insertion of 8 (based on dPCR nptII/SYS ratio = 4 for the ratio of the kanamycin resistance relative to the single homozygous gene copy systemin {SYS} gene). Although tissue was not available for dPCR of the second Ly60.2 transgenic, its T_1_ ‘selfed’ offspring provided a single gene copy hemizygous insertion (dPCR = 0.5). Simple protein extracts of the apoplastic transgenics displayed measurable mCherry fluorescence; therefore, this 96-well plate measurement was correlated with the subsequent dPCR. Self-pollinated hemizygous Ly60 T_2_ segregants displayed a Mendelian segregation (Fig. 5A).

This segregation also provided the homozygous segregants (dPCR = 1) as the highest expressing segregants (see Supplemental File C, Figure SC1). The analysis of multiple segregants from the hemizygous state provides a statistical measure of the variation in dPCR of +/- 0.16 as reflected by the dashed oval on Fig. 5A. This illustrates the tremendous discerning power of dPCR for gene segregation. We note, however, that achieving this required gaining experience in the processing of the samples for the dPCR method.

**Fig. 6:**
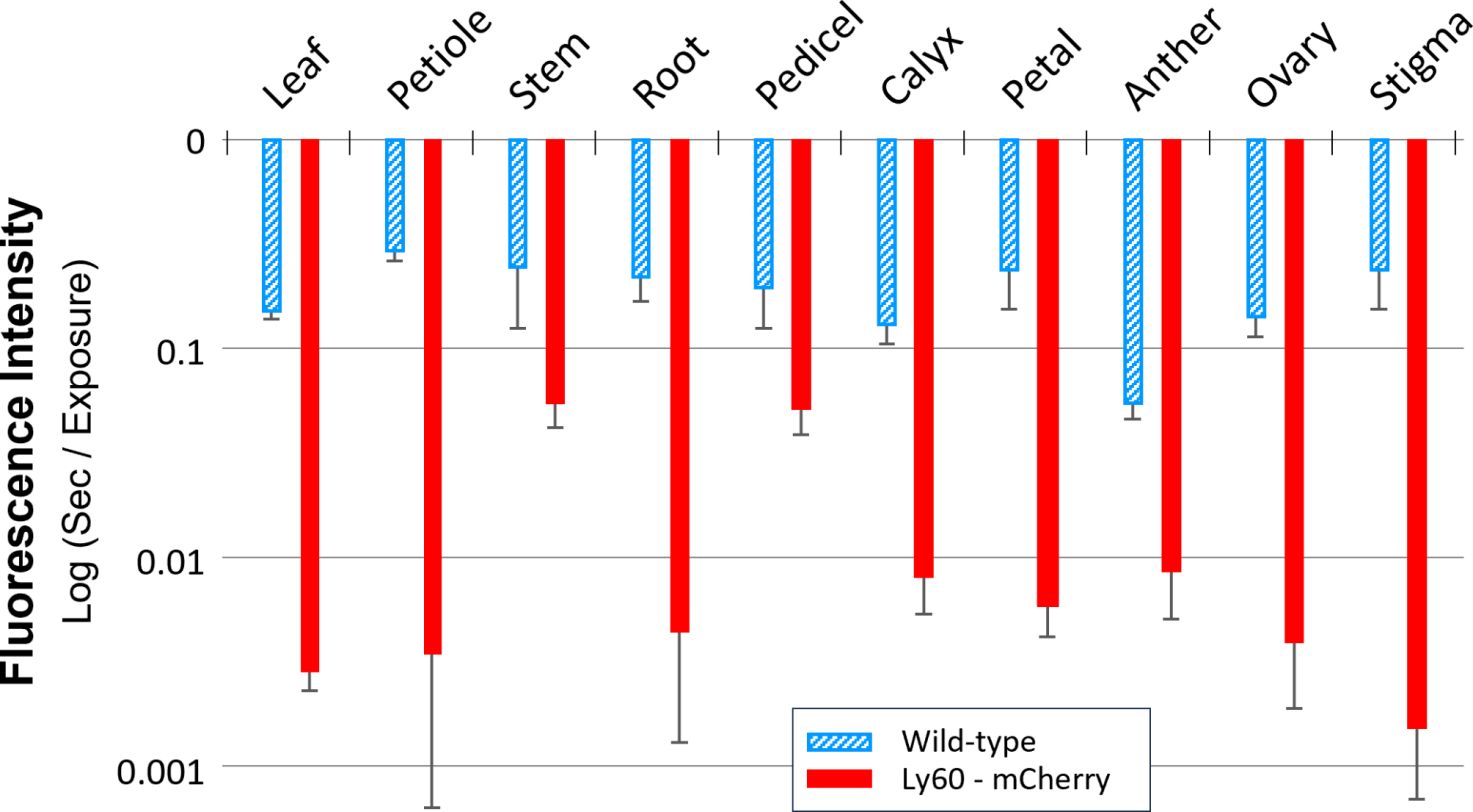
Characterization of Ly60 homozygous transgenic apoplastic mCherry fluorescence in various tissues relative to corresponding tissues in wild-type tomato. Fluorescence quantification based on incipient saturation of confocal imaging. Standard deviation bars based on 4–7 independent measurements

The incipient saturation confocal imaging method for characterizing the intensity of fluorescence described in methods provides a basis for a quantitative comparison of the Ly60 expression levels of mCherry in different tissues (Fig. 6). Noting the logarithmic scale, the expression levels of mCherry expression (measured as very short exposure times) are orders of magnitude higher than background autofluorescence in all tissues tested. Essentially no background autofluorescence is observable at the same level of exposure used for the transgenic Ly60 (Supplemental File C, Figure SC1). The distribution of mCherry expression can be observed from the associated Ly60 tissue images (Supplemental File C, Figure SC2) such as elevated expression towards the end of the stigma and in the immature tomato eggs of the ovary. The preferential root expression in the central procambium core is consistent with higher CaMV 35s promoter activity in these meristematic regions. Whereas apoplastic mCherry expression tends to elevate fluorescence in all tissues, the assessment of autofluorescence in wild-type tissues is instead biased by the localized autofluorescent structures such as vascular elements and epidermal structures (Supplemental File C, Figure SC3 and Figure SC4). Therefore, the quantitative difference between transgenic Ly60 and wild type autofluorescence background is likely underestimated.

The resource-consuming effort of segregating the Ly60 plants (> 100 plants, several thousand seeds over 3 years) was important in achieving reliable conclusions. Initial studies with sucker clones of the T_0_ apoplastic transformants as compared to wild-type controls suggested there was both observable whitefly uptake of mCherry, as well as mCherry uptake during egg expansion and entry through the pedicel. Subsequent efforts revealed numerous variations in autofluorescence from both the tomato and the whitefly. Tomato plant autofluorescence increases substantially with age as well as significant dependence on the light and growth conditions. There is also considerable variability in whitefly autofluorescence and highly localized fluorescence ‘hot spots’ (Supplemental File C, Figure SC12). We also observed silencing of expression in serial-propagated sucker clones to levels statistically indistinguishable from wild type (T-test using the incipient saturation quantification; compare Supplemental File C, Figure SC7), therefore it was important to be able to work with young homozygous plants.

**Fig. 7:**
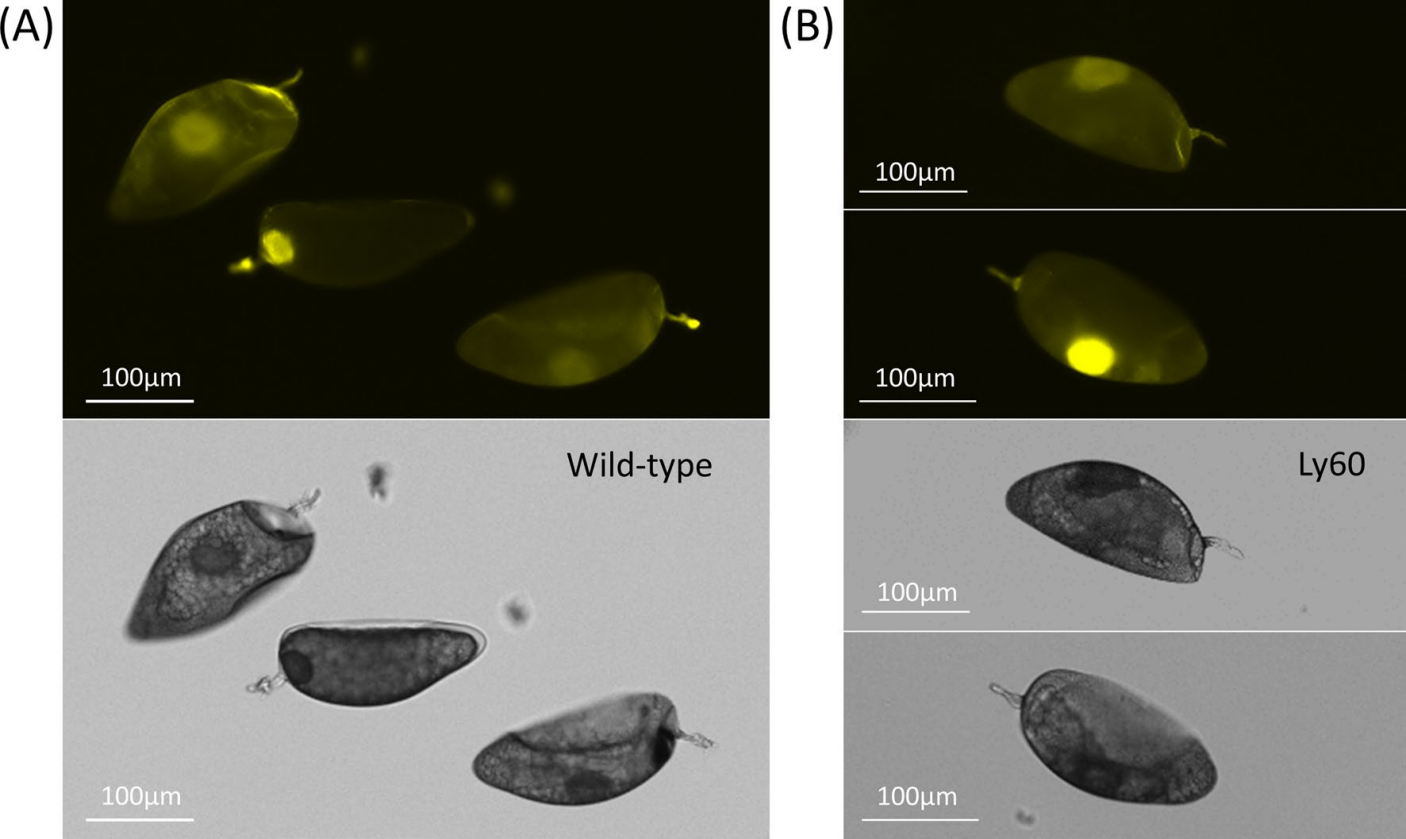
Comparison of fluorescence from eggs after a 48 h period of feeding on (**A**) wild-type controls vs. (**B**) transgenic Ly60 plants that were confirmed to have high-level apoplastic expression by confocal microscopy of the abaxial leaf surface. Fluorescent images taken at 1/3 s exposure time (upper) with the corresponding bright-field images below

The successful segregation of homozygous Ly60 line provided for systematic studies relative to wild-type control plants grown with replication under the same growth conditions. Whiteflies feeding on the homozygous Ly60 for 2–3 days did not display fluorescence that was elevated relative to whiteflies feeding on wild-type control. In addition, protein extracts of these whiteflies did not display elevated 620 nm fluorescence (580 nm excitation) relative to wild-type control (Supplemental File D). An examination of whitefly eggs laid on either wild-type tomato or cabbage by females previously feeding on mCherry-expressing Ly60 seedlings also did not display mCherry fluorescence that was significantly different from control. Having homozygous Ly60 seedlings easily screened to confirm lack of silencing also allowed for a systematic assessment of egg uptake of mCherry from the apoplast. As presented in Fig. 7, there is no consistent difference between the eggs recovered from Ly60 tomato plants as compared to wild-type control. There is a notable autofluorescence observed in the egg pedicel which could be from either the female colleterial gland secretion during pedicel insertion or in combination with plant defense (e.g. suberization) and tearing away some of the plant epidermis when the eggs are removed. Observations of attached eggs are challenging to interpret since the pedicel appears to behave as a ‘light guide’ from attached fluorescing plant tissues (Supplemental File C, Figure SC8).

### Phloem-specific ovary targeting transgenic characterization

The *Agrobacterium* transformation of roughly 100 tomato cotyledon explants with the Ly62 phloem-specific construct produced three first generation (T0) regenerated transgenics that produced T1 seed. Tissue was only available for Ly62.1, which indicated a 4 gene copy insertion (dPCR ~ 2) and was subjected to subsequent segregation to produce T1 segregants ranging from dPCR = 1 to 3.5. In contrast to the apoplastic expression, protein extracts of the phloem-specific expression were not sufficient to observe mCherry expression above background. The inability to observe mCherry in leaf extracts is not surprising given the highly phloem-localized expression driven by the CoYMV promoter (Figs. 4E and 5B and Supplemental File C, Figures SC9, SC5 & SC6). In contrast to protein extracts, confocal microscopy can readily discern the loss of expression for dPCR = 0 segregants (Fig. 5B). As we have experience with tomato phloem exudate proteomics [43], phloem exudate collection was utilized to attempt to observe mCherry expression in these transgenics but were similarly unable to discern statistically significant differences relative to wild-type tomato controls. A comparison of the exposure times in Fig. 4B (1/300 s) and **3E** (1/20 s), for homozygous Ly60 and Ly62 plants respectively grown under comparable conditions indicate that our phloem-specific expression is comparatively low relative to the apoplastic approach.

Despite lower Ly62 mCherry expression levels, the contrast provided by tissue specific expression in Ly62 provides clear observation of phloem-associated guard cells in all tissues tested. The stem and pedicle (flower stem) display the morphological characteristic of two bands of phloem, where the inner and outer phloem straddles the xylem in both tissues (Supplemental File C, Figure SC5). In contrast, roots have a single phloem band. The placenta of the immature ovary displays a stellate pattern to nourish the developing seed (Supplemental File C, Figure SC6-A). The phloem is also evident in the central core of the anthers (Supplemental File C, Figure SC6-B), but a substantial autofluorescence in the mature anther middle layer and interlocular septum was observed in non-transgenic tissue (Supplemental File C, Figures SC7-C & SC3-C). This wild-type anther autofluorescence is noted to rapidly increase after tissue excision such that observations of anthers were not considered reliable for interpretation of mCherry expression. The observation of phloem in the petals and calyx-sepal are observed to be very accessible to whitefly feeding (Supplemental File C, Figure SC9), which may explain the tremendous affinity of whiteflies towards *Solanaceous* flowers (and yellow sticky cards).

As with the apoplastic constructs, the effort of generating homozygous state (> 100 plants) was important for achieving consistent observations. Notably, the primary T0 Ly62.1 transformant was proliferated and cut back for over two years, where both the initial transformant, and its subsequent sucker clones displayed diminished phloem-specific expression that was comparable to background wild-type autofluorescence as assessed by confocal microscopy (Supplemental File C, Figures SC7 & SC9). This indicates that these transgenes experienced gene silencing despite the localized expression directed by the CoYMV promoter. Seedlings initiated from these silenced plants displayed the phloem-specific expression pattern, suggesting this gene silencing is not heritable. We are following up this observation with grafting and DNA demethylation treatments to better understand the nature of this silencing. There is concern that the dwarf tomato phenotype used in this work might be particularly prone to gene silencing. Using homozygous Ly62 seedlings that were confirmed for phloem-specific expression by microscopy, we were still not able to observe significant mCherry fluorescence above background wild-type controls in either the feeding adults or eggs laid on the Ly62 transgenics or on cabbage after Ly62 tomato feeding.

### Whitefly egg lysate accelerate mCherry proteolysis

**Fig. 8:**
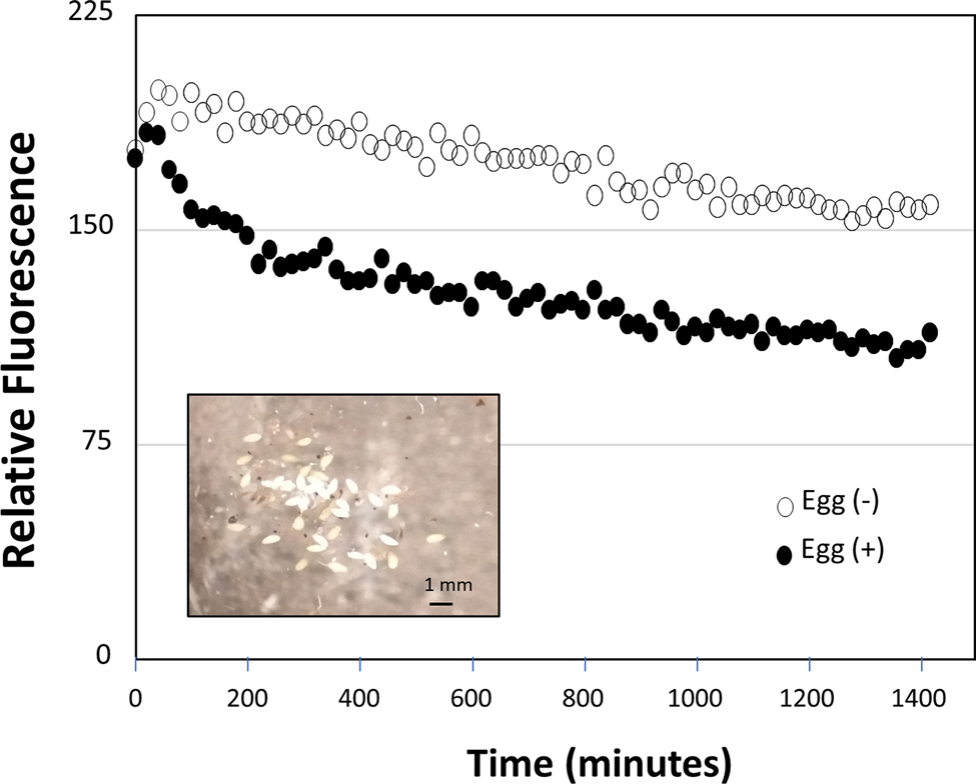
Assessment of protease activity in whitefly eggs measured as degradation of fluorescence of Ly60 mCherry leaf extract in a plate reader. Degradation with (solid) and without (open) macerated whitefly eggs. (Inset) Image of isolated whitefly eggs used in protease study

The lack of fluorescence in the eggs could be due to proteases within the embryo – including protection from insecticidal plant proteins. As a simple test for protease activity, 15 whitefly eggs collected from eggs laid over a 2–3 day period when egg expansion is greatest were crushed into a protein extraction buffer (without protease inhibitor) and added to an mCherry containing leaf extract from a Ly60 plant and compared to the treatment without egg extract (Fig. 8). The relative degradation rates over a 24-hour incubation demonstrate that the addition of eggs increased degradation of mCherry over the control from the Ly60 mCherry extract. This is consistent with a protease activity that might protect and even provide amino acids to the developing eggs.

### Preliminary studies of insecticidal chitinase

Transgenic tomato plants from the chitinase constructs underwent extensive study that is worth noting despite difficulties in providing conclusive insecticidal results. Interestingly, the two chitinase transgenics (Ly61 and Ly64) representing the C- and N- terminal protein transduction domains (PTD::SynVg: Chitinase / SynVg: Chitinase: PTD respectively) would not give meaningful dPCR results. Despite being able to PCR confirm the presence of both the chitinase and NptII kanamycin resistance gene (the dPCR target), the dPCR consistently failed more than a half dozen times during measurement groups where a dozen alternative transgenics being screened were successful. This inability to obtain either gene copy insertion or subsequent segregation led us to trying to use lateral branch sucker clones for experimentation on whitefly fecundity. Preliminary studies with multiple clones in a single cage analogous to our work on whitefly fecundity on cabbage [27] were promising; however, studies carried out with serially proliferated sucker clones in individual smaller cages and smaller numbers of flies for inoculation proved to be problematic. Besides introducing the potential for loss of chitinase expression due to silencing (as observed for mCherry in this work), our tomato sucker clones developed aberrantly compact growth structure and resulting dense trichomes relative to typical tomato seedlings which increased variability in the number of whiteflies that would survive inoculation (Supplemental File C, Figure SC10-C).

A considerable effort was made to move towards phenotypic characterization of chitinase activity using a *Rhizoctonia solani* fungal growth inhibition bioassay that was refined to include quantification via image analysis [45]; however, this effort also revealed a high variability in response to tomato protein extracts and phloem exudates. The fern chitinase was chosen based on its effectiveness in transgenic cotton [39]. Tomato has several dozen chitinase-related genes identified which are phylogenetically distant and primarily annotated as exo-chitinases, not endochitinases (see Supplemental File F). It is also possible that whiteflies have adapted to the broad range of chitinases in tomato. Numerous studies were conducted to try to overcome the issue of sucker clone morphology as well as individual plant replicates for fecundity kinetics. As we could not obtain definitive conclusions from that effort, we provide some of the details of that work as a supplemental description (Supplemental File E).

## Discussion

Exploring insecticidal activity targeting egg viability, rather than the adult whitefly, represents a unique strategy for insecticidal transgenic plants. Plants and insects have co-evolved defense strategies targeting eggs in response to elicitor / effector signals associated with ovideposition, where whiteflies and other phloem-feeding insects have the opportunity for precision manipulation of plant responses through their saliva [46]. A recent report achieved reduction in whitefly fecundity by swapping the jasmonic and salicyclic acid responsive promoters identified during whitefly feeding [47]. In the current work, genetic tools of apoplastic and phloem-specific expression are successfully demonstrated using mCherry as the surrogate reporter gene. In retrospect, a reporter with fluorescence shifted more into the red spectrum would perform better for the insect microscopy (Figs. 2 and 7, Supplemental File C, Figures SC11 & SC12). Apoplastic and phloem-specific expression have been used for more direct insecticidal activity such as insect-specific toxin (e.g. spider, scorpion) and lectins [48]. Phloem specific expression has been particularly useful for whitefly [49, 50]. Whitefly feeding requires considerable effort of insertion of the stylet into the vascular system to access the phloem. As a result, the female will often lay its eggs in a circular pattern reflecting rotating while feeding (see Supplemental File C, Figure SC10-B). In addition, the entirety of the whitefly life cycle, including the developing nymph stages, also relies on phloem feeding which has been successfully targeted in whitefly [51]. The effectiveness of transgene expression in phloem guard cells can be expected to be dependent upon its release into the phloem. Some insights can be obtained from expression of the insecticidal proteins of ‘Bt’ (*Bacillus thuringiensis*) - arguably the most successful implementation of plant GMO technology due to its ability to safely target the destructive larval stage of insects. Studies of aphids feeding on corn concluded a near absence of Bt proteins in the phloem [52]. Since the CaMV 35s promoter is expressed in guard cells [53], absence of Cry proteins in the phloem must result from being larger than the 70-kDa size exclusion cutoff for transport from the guard cells into the sieve elements. Interestingly, *Arabidopsis* phloem contains extensive proteins with known plastid-targeting sequences [54], apparently insufficient to overcome the default transport pathway. This illustrates that a better understanding of phloem loading of proteins could enable alternative transgenic strategies. Apoplastic mCherry (~ 26 kDa) can be expected to enter the phloem. Similarly, our 881-bp PTD-SynVG-mCherry fusion (~ 32.3 kDa) and the 861-bp PTD-SynVG-Tma12 chitinase (~ 31.9 kDa) would be expected to pass into the phloem. The majority of spider venoms are very small (2.5–5 kDa) [55] and should readily confer phloem feeding insect resistance. However, this introduces a significant challenge to public perception for plants expressing toxins, even if their activities do not have human off-target effects.

Our current research focused on the phloem-feeding whitefly because it is an efficient transmitter of plant viruses [56]. In conjunction with this effort was the hope of developing conditional lethals that would result in insect death in the absence of a culture environment suppression of a lethal gene. Notably, this may now be possible based on our recent development of in vitro axenic whitefly tissue culture and membrane feeding methods [4]. Since a goal of the plant protection application required viability of the adult whitefly as a vector for viral vector-based gene delivery, our assessment of insecticidal transgene expression targeted egg viability rather than adult toxicity. High levels of apoplastic mCherry reporter gene expression permitted transgene segregation analysis based on simple protein extracts. This expression approach benefited from enhancements embodied in the pEAQHT vector which includes the (511 bp) 5’-UTR enhancer of the cowpea mosaic virus, as well as the co-expression of the potyvirus anti-gene silencing P19 protein [57]. Given that there are extensive biochemical activities outside the plant cell, our use of LeXHT1 export signal peptide to achieve apoplastic translocation can be considered representative of many different alternatives. Phloem-localized expression of mCherry in companion cells could be monitored based on confocal microscopy where the overall total expression is minimized by using tissue specific promoters. This monocot virus promoter discovered over 30 years ago, has received little attention, but has been demonstrated to drive phloem-specific expression in tobacco [58]. In this current work, we had not utilized enhancements of phloem-specific expression such as co-expression of P19 and UTRs that would be worthy of inclusion. The rate of mCherry proteolysis in the whitefly egg protein extract was observed to be quite slow; therefore, these results do not preclude an effective egg-targeted insecticidal protein strategy for whitefly. The demonstration of laccase as a whitefly protective effector, and elevated activities of this protective enzyme in eggs [59] could be a CRISPR target to reduce the insects defense against plant-expressed insecticidal strategies. Leveraging existing ovicidal plant strategies should also be considered to provide multiple layers of protection [60].

A specific issue for the current work with transgenic tomato is that tomato has extensive insecticidal characteristics that may mask the utility of alternative strategies. The physical line of defense of trichomes and their associated phytochemicals [61] was noted for the sucker clones (and has also been observed to be a significant issue for the stunted growth phenotype of tissue culture environment [4]). In addition, extensive chitinase repertoire of tomato may reflect its tropical origin of domestication (see Supplemental File F). By comparison, cotton originated in the Nile river valley. Thus, the observation of improved whitefly resistance for transgenic cotton expressing tropical fern (*Tectaria macrodonta*) chitinase Tma12 [39] may represent the right match of separate evolutions between plants and their pests. Consistent with this observation, while cotton was protected from whitefly using a fern chitinase [39], corn has had insecticidal protection from corn borer by introducing a chitinase from the pest cotton leaf worm [62].

This overall effort illustrates the value of creating stable homozygous lines, as the combination of artifacts (age dependent plant and whitefly fluorescence, sucker clone morphology, gene silencing, etc.) would have led us to alternative (incorrect) conclusions if we had prematurely published these results. Digital PCR proved to be invaluable in this effort and could be used more prudently in genetic selection [63]; however, this core facility capability is planned to be abandoned at our major agricultural University due to lack of users. This may reflect the frequent characterizations of primary transformants in response to pressure for publication productivity. We were not able to determine the reason for dPCR failure for our chitinase transgenic tomato, but it is clear that homozygous lines could have avoided the problems created by attempts to use sucker clones. Similarly, the likelihood of gene silencing is greatly increased for multi-gene copy insertions and clones, which is far harder to assess for protective genes than for the reporter gene constructs emphasized in this work. An effort to outsource Southern blot analysis for gene copy number was not successful, but would be needed to overcome the multiplicity of issues presented by meristem cloning.

Although we were unable to demonstrate egg-targeting insecticidal plants with these studies, considerable progress towards localized gene expression was achieved. In particular, in vitro membrane feeding of insecticidal proteins to target phloem-feeding insects is a logical next step. Fluorescent reporter genes shifted more strongly into the red spectrum would provide better visualization in the insect and eggs although this would deteriorate visual screening in plants. An enzymatic reporter choice such as NanoLuc luciferase [64] could take advantage of extremely high signal-to-noise ratio of bioluminescence while keeping the size of the reporter gene to a minimum. The observations of gene silencing in older tomato plants for both the phloem-specific and apoplastic suggests that the co-expressed anti-gene silencing element P19 was not sufficient to overcome this issue. The homozygous lines of these transgenics would have utility in follow-up studies to better understand gene silencing mechanisms due to the ease of tomato grafting. The value and challenge of working with a crop plant, instead of model systems like *N. benthamiana* or Arabidopsis as well as the complexity required to work with insects are appreciated from this effort.

## Conclusions

The molecular tools for achieving both apoplastic and phloem-specific expression of insecticidal proteins are well developed. mCherry provides a reasonable imaging compromise for avoiding autofluorescence in both tomato leaf and whitefly, with eggs displaying rapid change in autofluorescence after being laid. A high signal-to-noise bioluminescent reporter could better answer functional protein delivery to eggs but lose the valuable fluorescence imaging. Digital PCR is a robust means of achieving tomato segregation to the single gene copy homozygous state which is important for consistent fecundity studies. Given the significant water uptake by the egg from the host plant, it is not surprising that proteolytic activity has evolved to protect the egg from this route of protein delivery, and choice of the protein to avoid that proteolysis would be a key component for that insecticidal strategy. Although we were not able to demonstrate protein delivery from the hemolymph, the phloem-feeding nature combined with vitellogenin ovary targeting may have promise for disrupting fecundity. A more systematic assessment of efficacy of this novel approach could be enabled by artificial diet feeding. The recently developed acyl-sugar knockout of wild tobacco (*Nicotiana benthamiana*) would provide significant advantages for further development of this insecticidal strategy for whitefly, including more direct targeting of the adult flies. While the applied nature of the funding for this research dictated many of the decisions in developing these approaches, the stage is now set for a more systematic assessment of the muti-faceted requirements (expression levels, dosage, fusion potency, proteolysis, etc.).

## Electronic supplementary material

Below is the link to the electronic supplementary material.

Supplementary Material 1: Supplemental File A: Signal Peptide mCherry Fusions

Supplementary Material 2: Supplemental File B: Design of the Synthetic Vitellogenin for Receptor-mediated Endocytosis

Supplementary Material 3: Supplemental File C: Additional Supporting Confocal Images / Figures

Supplementary Material 4: Supplemental File D: Examination for Whitefly Feeding on mCherry Transgenic Tomato

Supplementary Material 5: Supplemental File E: Tomato Transgenic for Tma12 Chitinase

Supplementary Material 6: Supplemental File F: Native Chitinase Genes in Tomato

Supplementary Material 7: Supplemental File G: Additional Cloning Information / Constructs

## Data Availability

In addition to supplementaty information files, additional details of tomato transformation narratives and segregation spreadsheet logs as well as data associated with manuscript figures including fluorescence imaging exposure time are available from datacommons@psu.edu, at the specific URL: 10.26208/V1C6-5E75.

## Abbreviations

IAA: Indole acetic acid
GM: Genetically modified
MS: Murashige & Skoog
PTD: Protein transduction domain
SynVg: Synthetic vitellogenin domain
Vg: Vitellogenin
VgR: Vitellogenin receptor
