# Supplementary material for: Transgenic tomato strategies targeting whitefly eggs from apoplastic or ovary-directed proteins
Source: BMC Plant Biol. 2024 Dec 27;24:1262. doi: 10.1186/s12870-024-05852-5 (PMC11673810; doi:10.1186/s12870-024-05852-5)
Supplement: Supplementary file 1 — Supplementary Material 1: Supplemental File A: Signal Peptide mCherry Fusions [file 12870_2024_5852_MOESM1_ESM.docx]

**Supplemental File A – Signal Peptide mCherry Fusions**

The signal peptides of several proteins known to have extracellular function were evaluated using the SignalP 5.0 tool (<https://services.healthtech.dtu.dk/service.php?SignalP-5.0>), and the results are denoted in Table A1 below.

**Table A1: Signal Peptide Search**

| **Gene** | **Gene ID**  **UniPROT** | **AA** | **Y-**  **score** | **D-**  **score** | **Signal Peptide / Comment** |
| --- | --- | --- | --- | --- | --- |
| PLA1 | [101251307](https://www.ncbi.nlm.nih.gov/gene/101251307)  (119-643bp) | 24 | 0.868 | 0.887 | MAVFSRNVFVMAILFFSLLSFTEA  Embryogenic Phytocyanin-Like Arabinogalactan homolog; Early nodulin-like protein 3 (ENOD3) |
| GP1 | [543991](https://www.ncbi.nlm.nih.gov/gene/543991)  [Q40161](https://www.uniprot.org/uniprotkb/Q40161) | 27 | 0.588 | 0.759 | MHTKIHLPPCILLLLLFSLPSFNVVVG  Polygalacturonase-1 non-catalytic subunit beta  Limit solubilization of cell wall polyuronides during ripening |
| XTH1 | [544272](https://www.ncbi.nlm.nih.gov/gene/544272)  [Q40144](http://www.uniprot.org/uniprot/Q40144) | 22 | 0.497 | 0.727 | MGIIKGVLFSIVLINLSLVVFCG  Probable xyloglucan endotransglucosylase/hydrolase 1  Cell wall construction |
| STIG1 | [543904](https://www.ncbi.nlm.nih.gov/gene/543904)  [Q6EEH1](https://www.uniprot.org/uniprotkb/Q6EEH1) | 23 | 0.495 | 0.659 | MDFIILLIAILALSSTPITIISG  Signal for stigmatic exudate |

All of these signal peptides were fused to mCherry reporter gene via overlap-extension PCR; however, time constraints required prioritization in subsequent transformation, regeneration and characterization. The prioritization was based on rating the signal peptides by the Y-score (combined cleavage score), which predicts whether the region is actually a cleavage site, and the D-score (discrimination score), which discriminates between cleavage sites and non-cleavage sites. The PLA1 protein signal peptide had the highest rating and was therefore a priority candidate (however transformation events failed to give transgenics). Although GP1 had a high rating its specialized functions in fruit ripening and pollen tube growth seemed less general than a cell wall enzyme. This lead to use of XTH1 signal peptide as a second priority which was ultimately successful in the generation of transgenic tomato.
