# Supplementary material for: Transgenic tomato strategies targeting whitefly eggs from apoplastic or ovary-directed proteins
Source: BMC Plant Biol. 2024 Dec 27;24:1262. doi: 10.1186/s12870-024-05852-5 (PMC11673810; doi:10.1186/s12870-024-05852-5)
Supplement: Supplementary file 2 — Supplementary Material 2: Supplemental File B: Design of the Synthetic Vitellogenin for Receptor-mediated Endocytosis [file 12870_2024_5852_MOESM2_ESM.docx]

**Supplemental File B – Design of the Synthetic Vitellogenin for Receptor-mediated Endocytosis**

The goal of the SynVG design is to provide a reasonably sized ovary-targeting sequence in whitefly based on literature and a bioinformatic analysis of the sequence of the native vitellogenin (**Vg**) gene in many species. There a numerus analyses of the vitellogenin protein and its associated receptor (**VgR**), including insect specific analysis ([REF1, REF2](http://journals.plos.org/plosone/article?id=10.1371/journal.pone.0155306), [REF3](http://www.genetics.org/content/127/4/769.short)). There are two protein domains that are typically found in Vg designated R/KXXR/K and a split domain DGXR … GL/ICG motifs. At the biomolecular level, this corresponds to facilitating the interaction between the SynVG protein domain, and the whitefly vitellogenin receptor (VgR). **Table B1** includes many of the sequences used for alignments.

**Table B1**: Bioinformatic References used in designing SynVG.

| **Species** | **Organism** | **Protein** | **Uniprot / GenBank** |
| --- | --- | --- | --- |
| Frog | *Xenopus laevis* | Vitellogenin-A1 | P19009 |
| Whitefly | *Bemisia Tabaci* | Vitellogenin | V9HZ11 |
| Honeybee | *Apis mellifera* | Vitellogenin | Q868N5 |
| Fruit fly | *Drosophila melanogaster* | Vitellogenin-3 | P06607 |
| Silk moth | *Bombyx mori* | Vitellogenin | Q27309 |
| Mosquito | *Aedes aegypti* | Vitellogenin-A1 | [Q16927](https://www.uniprot.org/uniprot/Q16927) |
| Fruit fly | *Ceratitis capitata* | Vitellogenin-1 | P27878 |
| Weevil | *Anthonomus grandis* | Vitellogenin | Q05808 |
| Puffer | *Takifugu pardalis* | Vitellogenin | A0A292G9J6 |
| Tilapia | *Oreochromis aureus* | Vitellogenin | AAD48085.1 |
| Prawn | *Macrobrachium rosenbergii* | Vitellogenin | BAB69831.1 |
|  |  |  |  |
| Whitefly | Biotype B | Vitellogenin | ADU04392.1 |
|  | Biotype Q |  | ADU04394.1 |
|  | Biotype ZHJ-II |  | ADU04393.1 |
|  | Aisa I |  | ANF29558.2 |
|  | MEAM1 | vitellogenin-like | XP_018897089.1 |
|  | MEAM1 | vitellogenin-A1-like | XP_018912902.1 |

**The R/KXXT/K motif** was explicitly given in Clawed Frog as RIIKSTDF ([REF](https://www.ncbi.nlm.nih.gov/pmc/articles/PMC5312104/#BIO022376C12)), where the corresponding motif in whitefly is quite divergent (DIVKTTNY) with the charged lysine is particularly important. This motif corresponds to amino acids (aa 212-220 / 239-246) at the N-terminal end of the two vitellogenin-like proteins in the MEAM1 assembly (see table B1).

**The DGXR … GL/ICG motif** is clearly apparent in the extensive alignment of insect vitellogenins as conducted by Tufail and Takeda ([REF](https://pubmed.ncbi.nlm.nih.gov/18789336/)) combined with additional molecular characterization ([REF](http://journals.plos.org/plosone/article?id=10.1371/journal.pone.0155306), **Figure 3**), led to the following canonical insect protein motif (DGARVKFQAASQYRGAVRCICG) hypothesized to form a beta-sheet structure that interacts with the VgR binding domain. The analogous Vg domain in our specific *Bemisia tabaci* MEAM1 was identified by BLASTing the Bemisia Asia strain sequence against the MEAM1 chromosome assemblies confirmed homology with Locus: XP_018897089, XP_018912902 which were aligned with other whitefly Vg genes:

DGARVKFQAASQYRGAVRGICG  MEAM1

DGARVKFQAASQYRGAVRGICG  MEAM1 alternative assembly

DGARVKFQAASQYRGAVRGICG  Asia

DGARIKIQAANQYRGAVRGMCG  biotype ZHJ-II

DGARIKIQAANQYRGAVRGMCG  biotype Q

DGARIKIQAANQYRGAVRGMCG  biotype B

This motif is towards the C-terminal of this rather large protein (aa 1871-1892 / 1998- 2019) in the two MEAM1 assemblies which presents a difficulty as it is not reasonable to have a more than 3Kb targeting sequence. As there is extensive research that involves more direct studies of binding, these were probed further with the goal of identifying a much smaller egg-targeting domain.

**Other Binding Studies**:

An extensive ligand binding analysis of the blue tilapia (*Oreochromis aureus*) vitellogenin sequence that identified an 84aa fragment that was sufficient for VgR binding (Li, Sadasivam, & Ding, 2003. DOI: <https://doi.org/10.1074/jbc.M205067200>). Notably, this fragment aligns towards the N-terminal of the whitefly Vg gene with the DIVKTTNY binding motif at its center. A follow-up sophisticated analysis of the prawn (*Macrobrachium rosenbergii*) Vg used peptide array to pull down and sequence protein interacting domains via mass spectrometry (Roth et al., 2013. DOI: <https://doi.org/10.1002/cbic.201300152>). This work concluded the prawn (237-260) sequence having a dominant role for Vg binding (DKNIIKPAYGSYKYVEAHQESVLR) which also corresponds to the N-terminal 84aa fragment of tilapia. Noting that a single point mutation in the tilapia Vg (K-185) dramatically attenuated VgR interaction (Li, Sadasivam, & Ding, 2003. DOI: <https://doi.org/10.1074/jbc.M205067200>), and the proximity of this lysine to this important binding fragment, it was considered important to include this lysine in the whitefly MEAM1 SynVG.

Align prawn fragment (DKNIIKPAYGSYKYVEAHQESVLR) to MEAM1 N-terminal

Prawn           D---KNIIKPA----------YGSYKYVEA------------------------------
MEAM1           DTQGQNLKKSSHNQLPKENKPYGVYKTMEDSVTGECETLYDVSPLPEITLQTKPWLVPFP
                *   :*: *.:          ** ** :*

Prawn           -----------------------------------------------HQESVLR------
MEAM1           NFRENGQFIDIVKTTNYSKCEARSAYHFGITGLTNWKPASNQMGQFLSRSNINRVVISGN

The remaining prawn MS fragments were also aligned with *Bemisia tabaci* MEAM1 and a breadth of the other Vg sequences noted above in an attempt to prioritize the Vg-VgR binding domains to include in the SynVg sequence.

N-Terminal Prawn Vg binding fragments:

237–260 DKNIIKPAYGSYKYVEAHQESVLR

377–386 VmVEELISGK

606–617 SRLETIVLPSNFTK

775–785 IDELINSLFDK

786–792 FDNMINR

824–833 LEGTAVAGIK

C-Terminal Prawn Vg binding fragments:

1066–1077 QVQMIAYFSESR
1444–1452 mVNPLSTLR
1861–1874 NAAASFIIENENQK

2447–2455 ASNLQELVK

It was noted that the none of the prawn protein fragments were ‘pulled down’ by a region corresponding to the C-terminal (DGAR…GMCG) motif. Therefore, although this motif is clearly highly conserved in whitefly, it was decided to prioritize the N-terminal DIVKTTNY motif based on these physical binding studies. A summary of various assessments of this N-terminal region of the Whitefly MEAM1 Vg is presented in **Figure B1**.


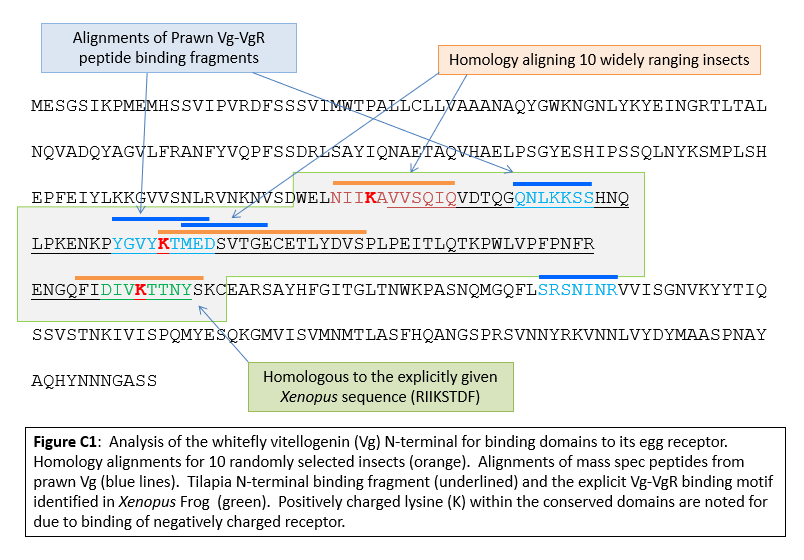


**Figure B1**: Analysis of the whitefly vitellogenin (Vg) N-terminal for binding domains to its egg receptor. Homology alignments for 10 randomly selected insects (orange). Alignments of mass spec peptides from prawn Vg (blue lines). Tilapia N-terminal binding fragment (underlined) and the explicit Vg-VgR binding motif identified in *Xenopus* Frog (green). Positively charged lysine (K) within the conserved domains are noted due to the binding of negatively charged receptor.

Noting a convergence of sequence homology and binding studies to this N-terminal regions, which also correspond well to the original 84aa amino acid motif of tilapia, an aggregate of this bioinformatic analysis was used to create a MEAM1 specific VgR-targeting sequence for the *in vivo* delivery of proteins to the whitefly eggs during female feeding on the transgenic plant.

The analysis led to three key whitefly domains in the MEAM1 Vg sequence:

- Motif 1: NII**K**AVVSQIQ
- Motif 2: QNL**KK**SS
- Motif 3: YGVY**K**TMEDSVTGECETLYDV
- Motif 4: QFIDIV**K**TTNY

The corresponding Vg targeting sequence (taking liberty to include the authors initials) is:

**W**ELNIIKAVVSQIQVDTQGQNLKKSSHNQLPKENKPYGVYKTMEDSVTGECETLYDVSPLPEITLQTKPWLVPFPNFRENGQFIDIVKTTNYSK**C**

This would result in a 95aa / 285nt protein tag which was considered to be excessively large for this initial test of transgene delivery. By retaining the two motifs corresponding to the canonical motifs R/KXXR/K and a split domain DGXR … GL/ICG, this targeting domain was condensed to:

WELNIIKAVVSQIQQNLKKSSYKTMEDSVTGECETLYDVSQFIDIVKTTNYSKC

With a tomato codon optimized sequence for SynVg of:

TGG GAA CTG AAT ATC ATT AAG GCT GTG GTG TCA CAA ATA CAA CAG AAT CTG AAG AAA

AGT TCC TAT AAA ACT ATG GAG GAC AGC GTC ACT GGA GAG TGC GAG ACA TTG TAC GAT

GTC AGC CAA TTT ATT GAT ATA GTC AAA ACA ACT AAT TAC AGT AAA TGC

The Drosophila protein transduction domain (PTD) is:

RQIKIWFQNRRMKWKK

This was tomato codon optimized to give:

AGG CAA ATC AAG ATT TGG TTT CAA AAC CGA AGG ATG AAG TGG AAA AA
