# Supplementary material for: Transgenic tomato strategies targeting whitefly eggs from apoplastic or ovary-directed proteins
Source: BMC Plant Biol. 2024 Dec 27;24:1262. doi: 10.1186/s12870-024-05852-5 (PMC11673810; doi:10.1186/s12870-024-05852-5)
Supplement: Supplementary file 3 — Supplementary Material 3: Supplemental File C: Additional Supporting Confocal Images / Figures [file 12870_2024_5852_MOESM3_ESM.pptx]

## Slide 1
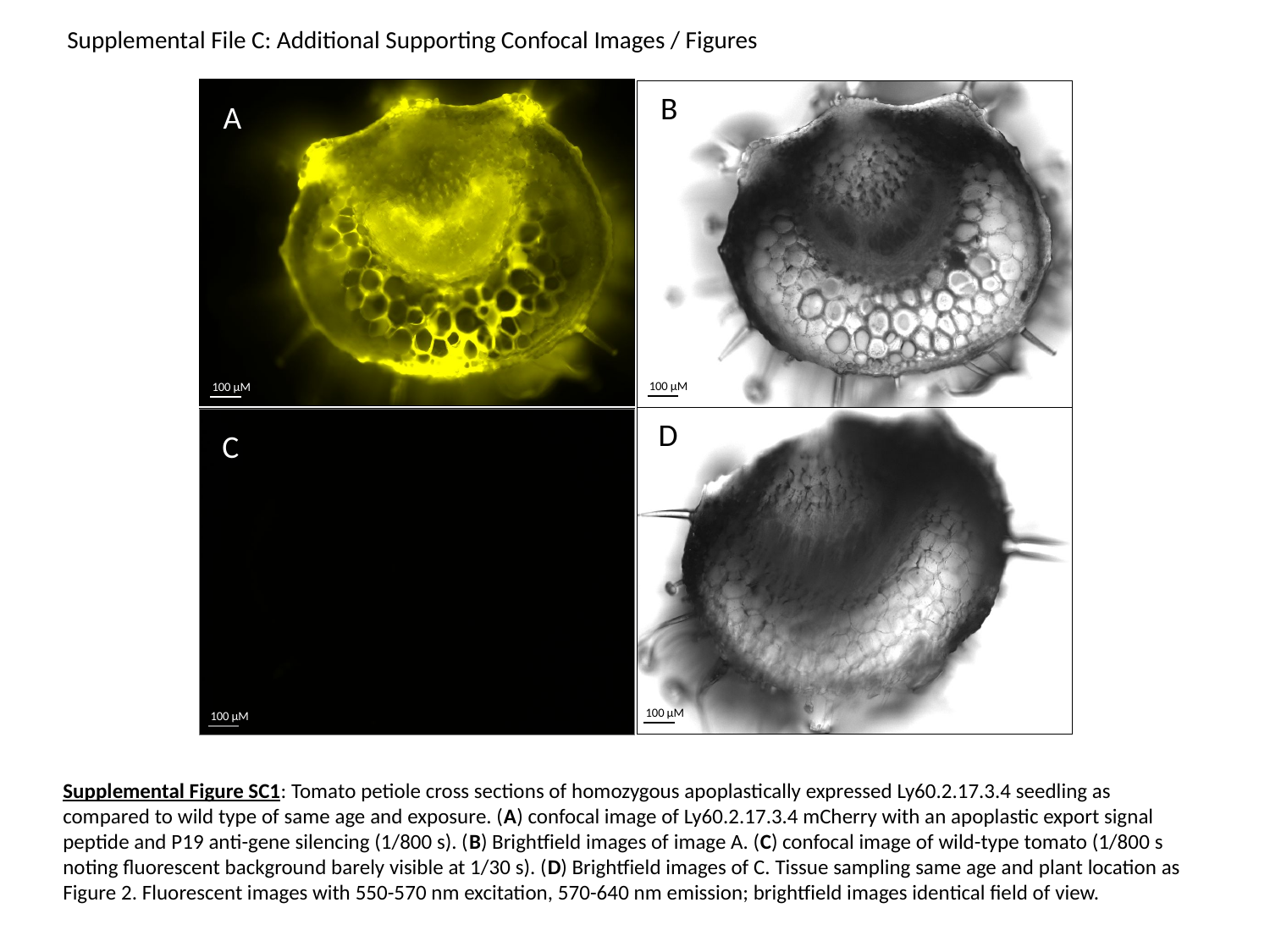

Supplemental File C: Additional Supporting Confocal Images / Figures
A
100 µM
B
100 µM
D
100 µM
C
100 µM
Supplemental Figure SC1: Tomato petiole cross sections of homozygous apoplastically expressed Ly60.2.17.3.4 seedling as compared to wild type of same age and exposure. (A) confocal image of Ly60.2.17.3.4 mCherry with an apoplastic export signal peptide and P19 anti-gene silencing (1/800 s). (B) Brightfield images of image A. (C) confocal image of wild-type tomato (1/800 s noting fluorescent background barely visible at 1/30 s). (D) Brightfield images of C. Tissue sampling same age and plant location as Figure 2. Fluorescent images with 550-570 nm excitation, 570-640 nm emission; brightfield images identical field of view.

## Slide 2
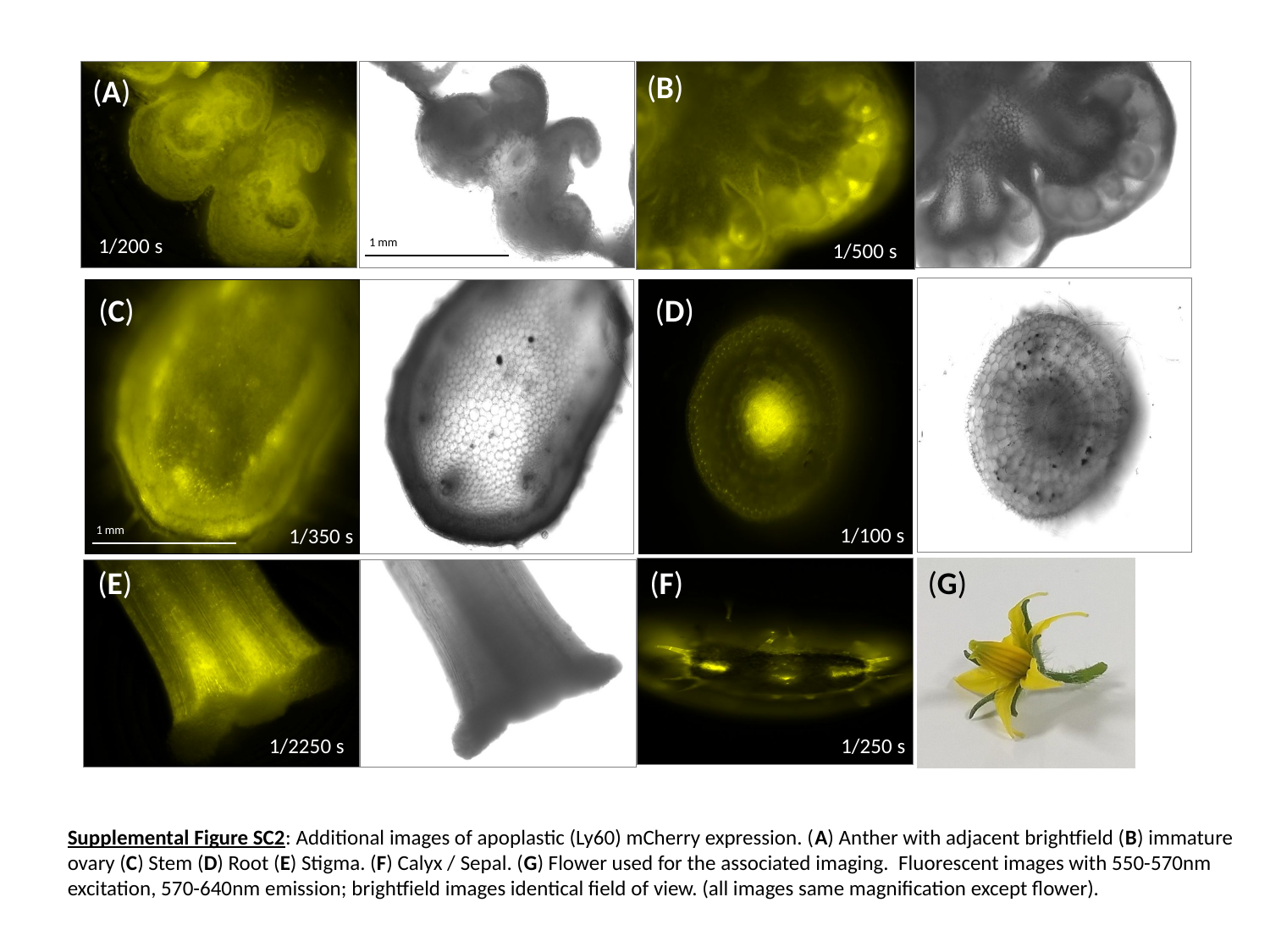

(B)
(A)
1/200 s
1 mm
1/500 s
(D)
Root
1/100 s
1/350 s
1 mm
(C)
(D)
(E)
(F)
(G)
1/2250 s
1/250 s
Supplemental Figure SC2: Additional images of apoplastic (Ly60) mCherry expression. (A) Anther with adjacent brightfield (B) immature ovary (C) Stem (D) Root (E) Stigma. (F) Calyx / Sepal. (G) Flower used for the associated imaging. Fluorescent images with 550-570nm excitation, 570-640nm emission; brightfield images identical field of view. (all images same magnification except flower).

## Slide 3
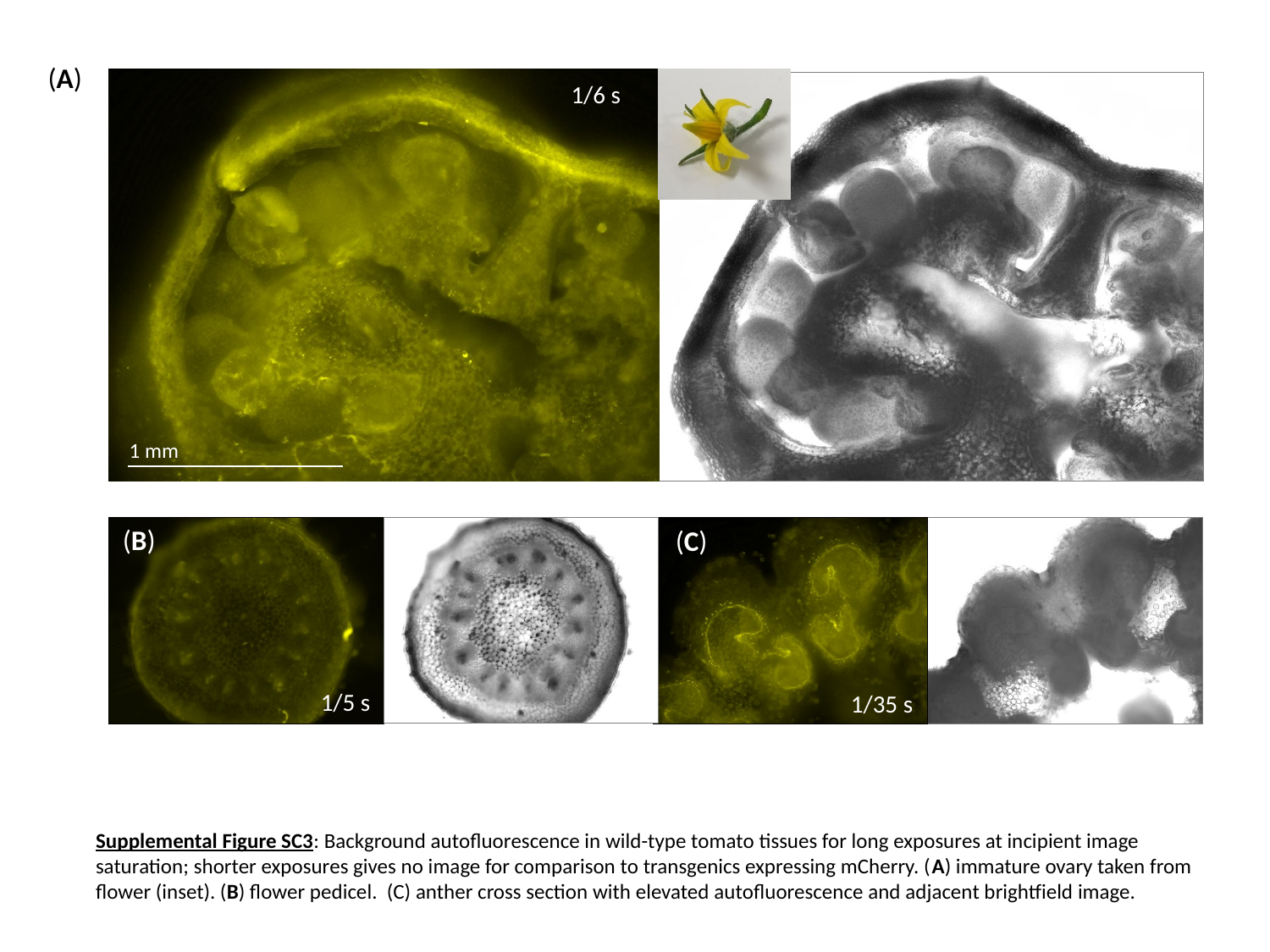

(A)
1/6 s
[8/20/23]
Immature Fruit Stem
1 mm
(B)
(C)
1/35 s
1/5 s
Supplemental Figure SC3: Background autofluorescence in wild-type tomato tissues for long exposures at incipient image saturation; shorter exposures gives no image for comparison to transgenics expressing mCherry. (A) immature ovary taken from flower (inset). (B) flower pedicel. (C) anther cross section with elevated autofluorescence and adjacent brightfield image.
1 mm
1/40s

## Slide 4
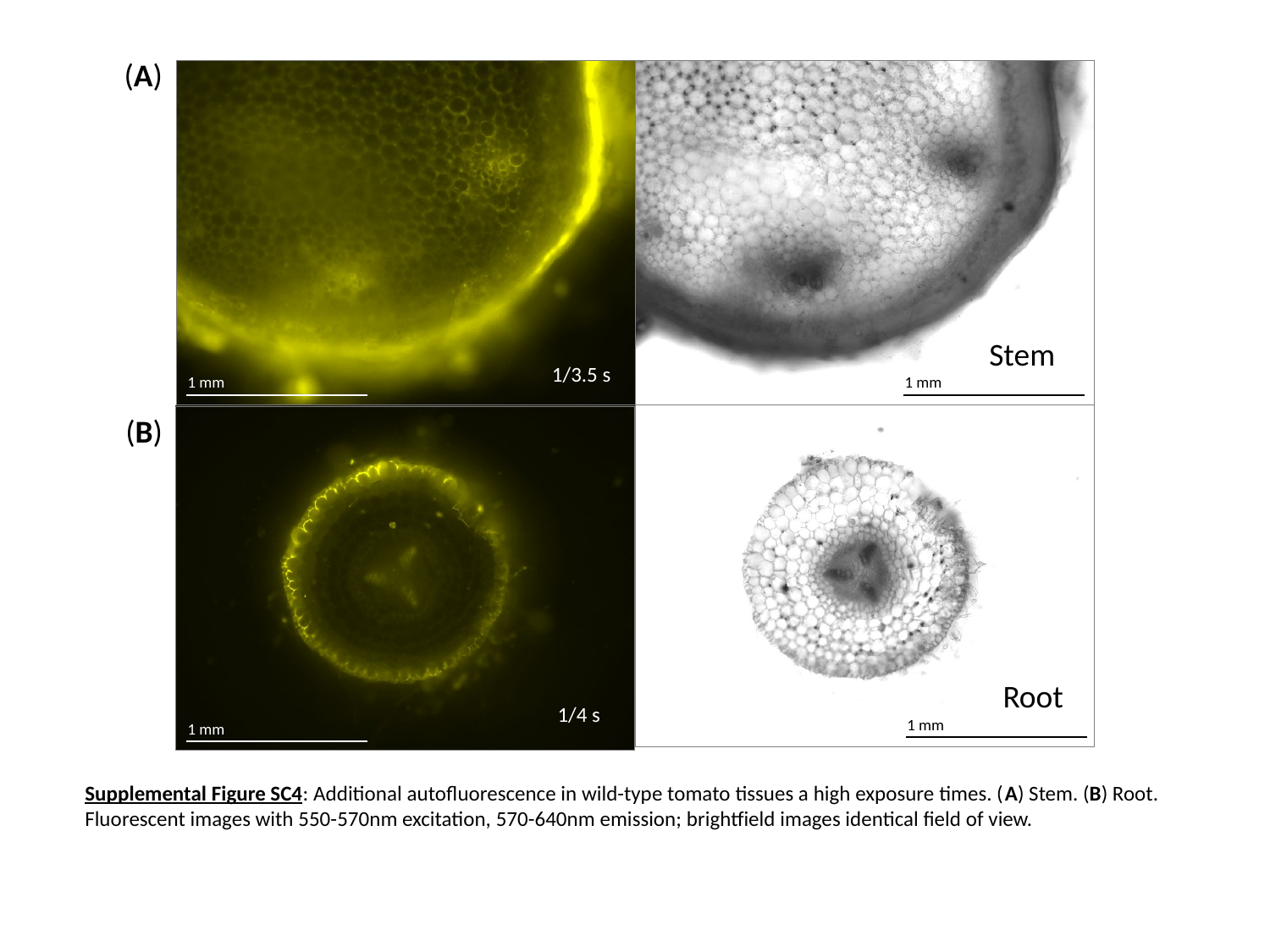

(A)
Stem
1 mm
1/3.5 s
1 mm
Root
1 mm
(B)
1/4 s
1 mm
Supplemental Figure SC4: Additional autofluorescence in wild-type tomato tissues a high exposure times. (A) Stem. (B) Root. Fluorescent images with 550-570nm excitation, 570-640nm emission; brightfield images identical field of view.

## Slide 5
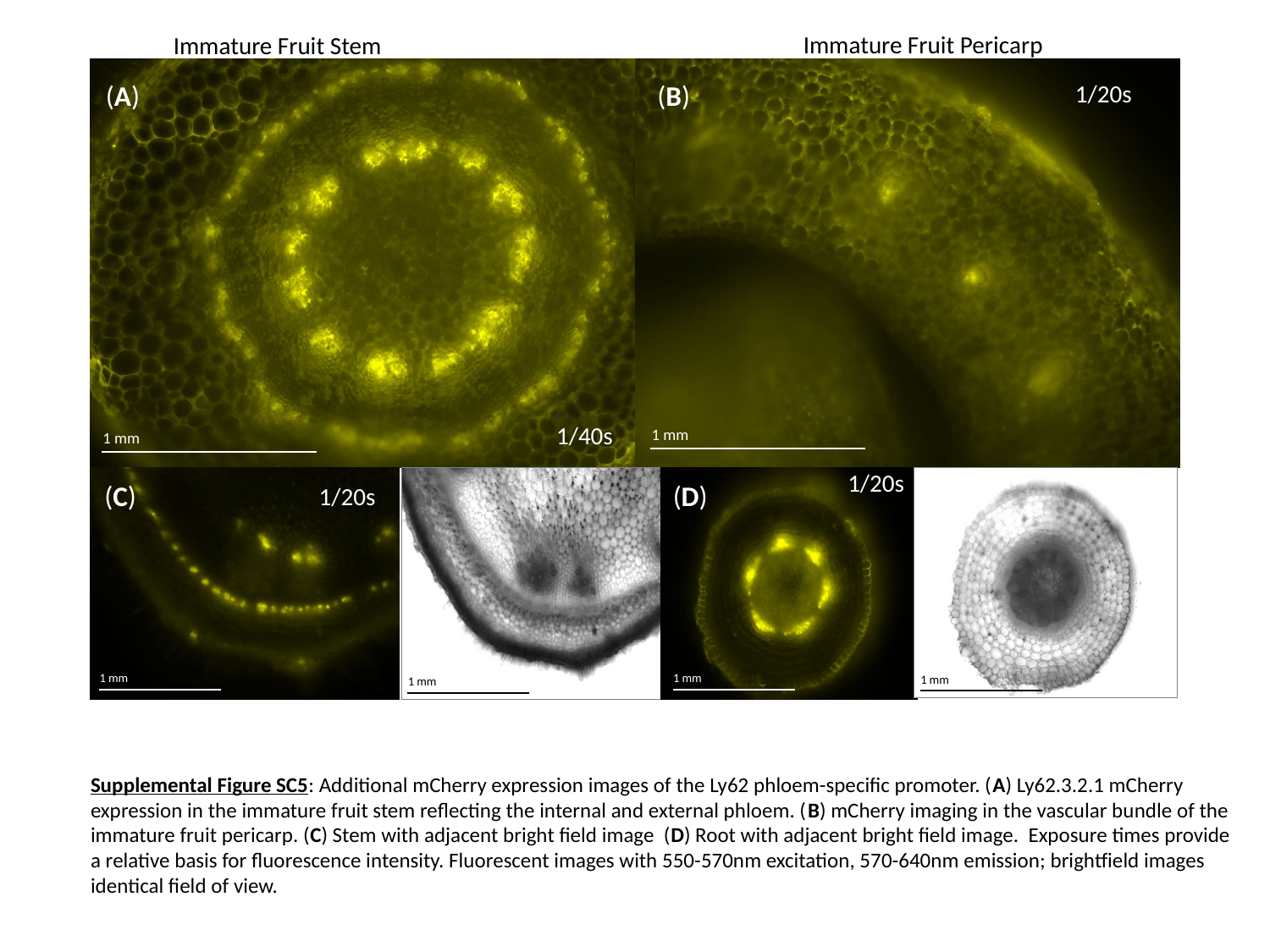

Immature Fruit Pericarp
Immature Fruit Stem
1 mm
(A)
(B)
1/20s
1/40s
1 mm
1/20s
(C)
(D)
1/20s
1 mm
1 mm
1 mm
1 mm
Supplemental Figure SC5: Additional mCherry expression images of the Ly62 phloem-specific promoter. (A) Ly62.3.2.1 mCherry expression in the immature fruit stem reflecting the internal and external phloem. (B) mCherry imaging in the vascular bundle of the immature fruit pericarp. (C) Stem with adjacent bright field image (D) Root with adjacent bright field image. Exposure times provide a relative basis for fluorescence intensity. Fluorescent images with 550-570nm excitation, 570-640nm emission; brightfield images identical field of view.
1 mm

## Slide 6
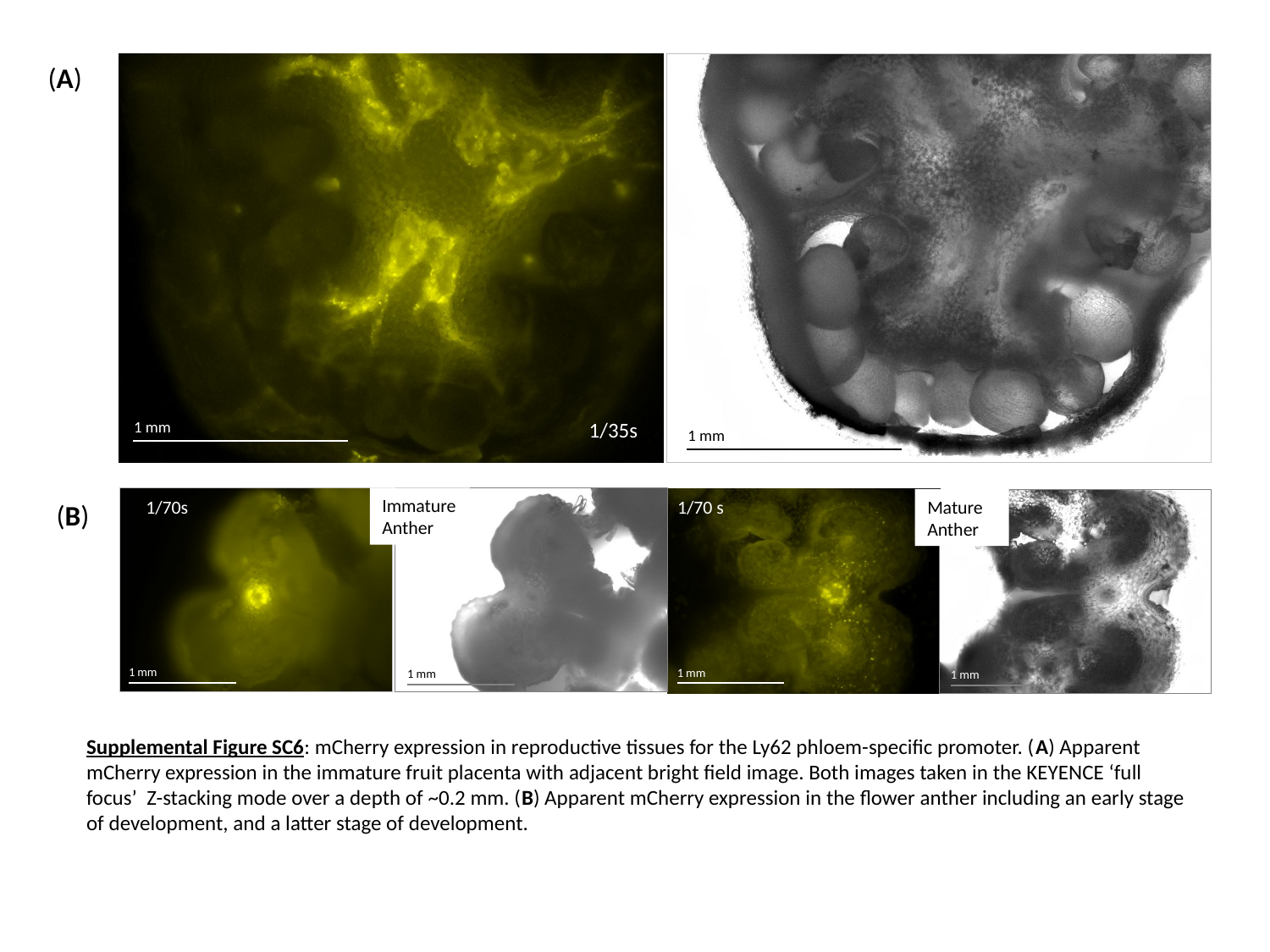

(A)
1/35s
1 mm
1 mm
Immature Anther
1 mm
1/70s
1 mm
1/70 s
1 mm
Mature Anther
1 mm
(B)
Supplemental Figure SC6: mCherry expression in reproductive tissues for the Ly62 phloem-specific promoter. (A) Apparent mCherry expression in the immature fruit placenta with adjacent bright field image. Both images taken in the KEYENCE ‘full focus’ Z-stacking mode over a depth of ~0.2 mm. (B) Apparent mCherry expression in the flower anther including an early stage of development, and a latter stage of development.

## Slide 7
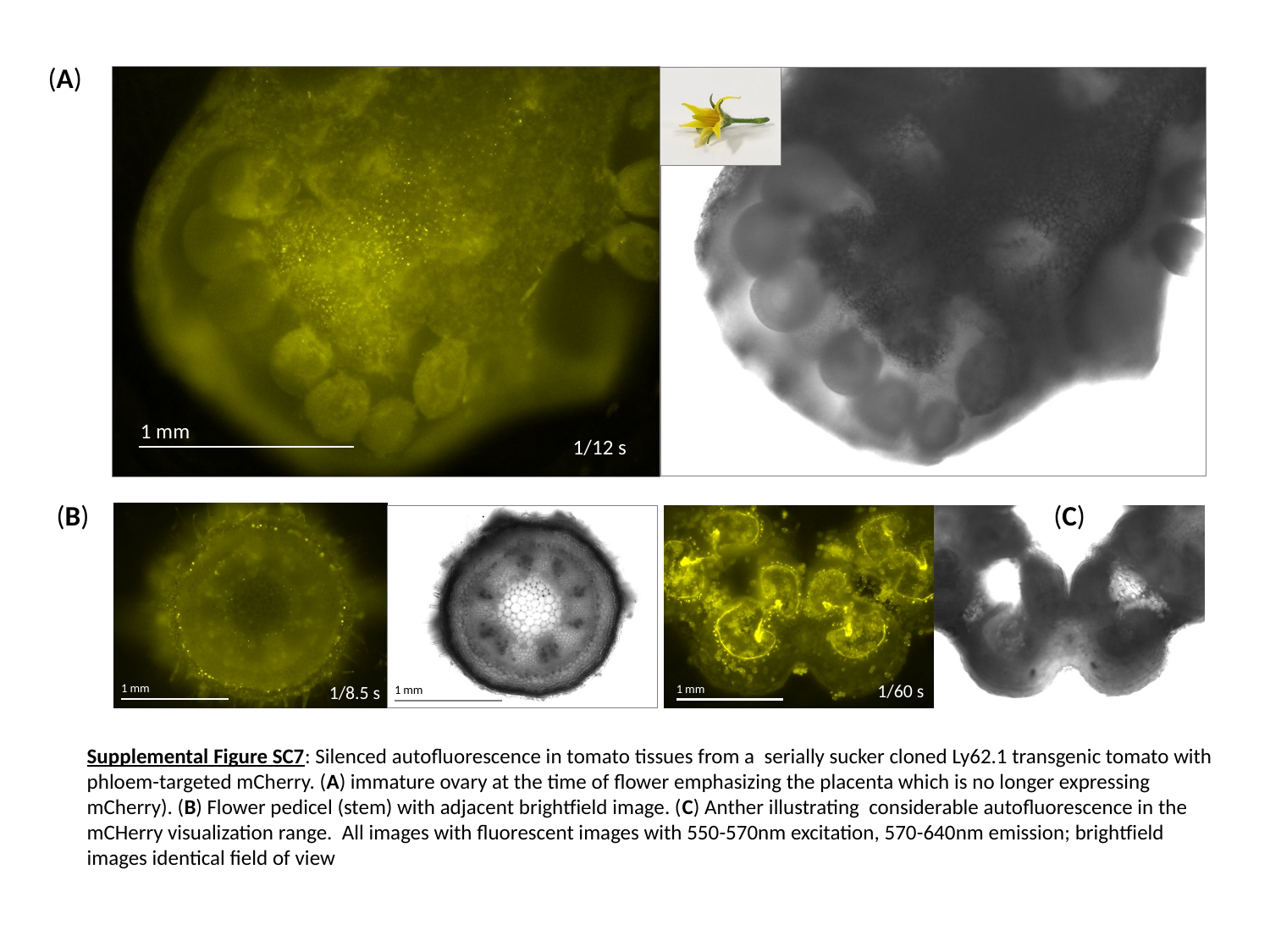

(A)
1 mm
1/12 s
(B)
(C)
1/60 s
1/8.5 s
1 mm
1 mm
1 mm
Supplemental Figure SC7: Silenced autofluorescence in tomato tissues from a serially sucker cloned Ly62.1 transgenic tomato with phloem-targeted mCherry. (A) immature ovary at the time of flower emphasizing the placenta which is no longer expressing mCherry). (B) Flower pedicel (stem) with adjacent brightfield image. (C) Anther illustrating considerable autofluorescence in the mCHerry visualization range. All images with fluorescent images with 550-570nm excitation, 570-640nm emission; brightfield images identical field of view

## Slide 8
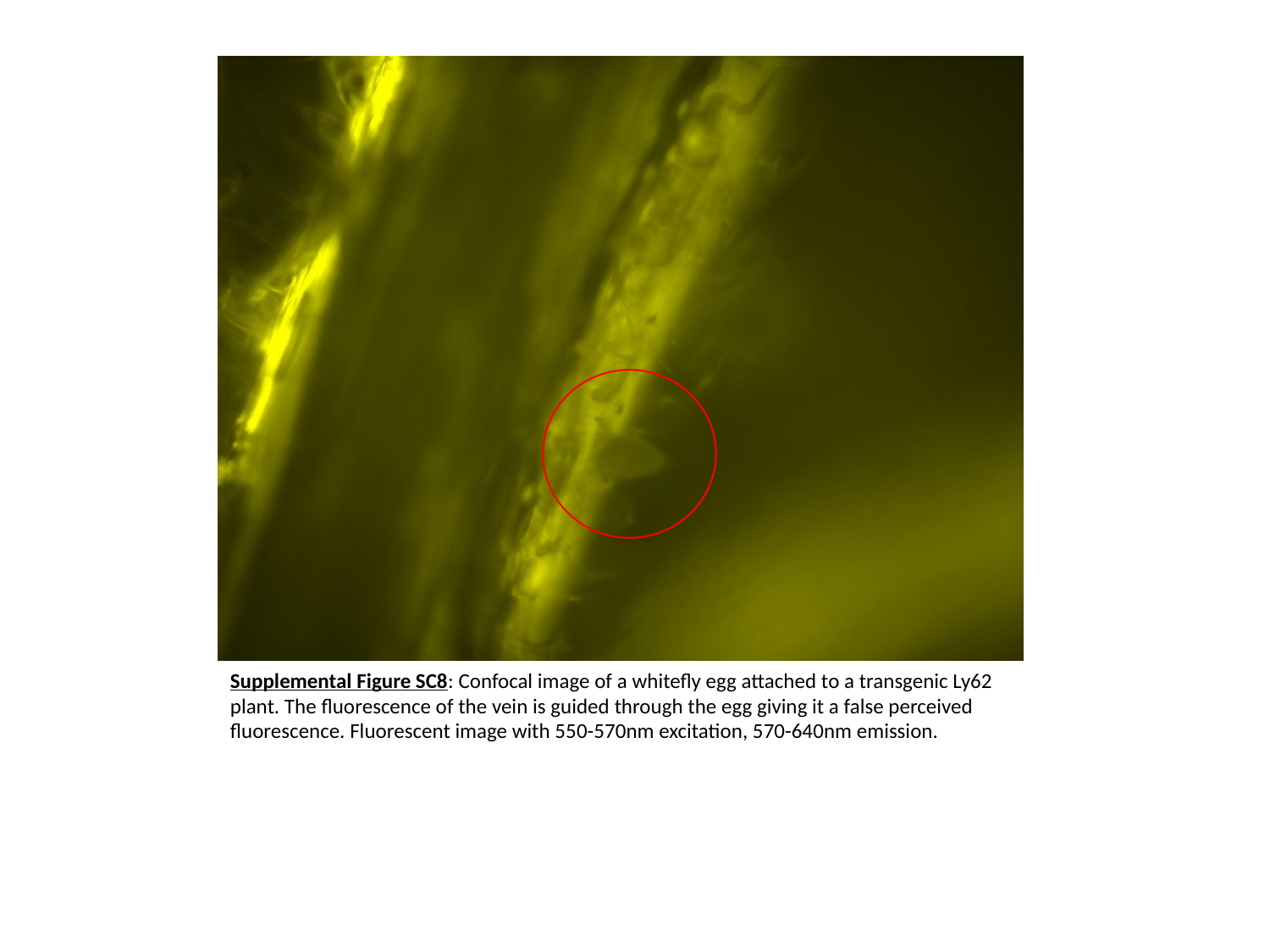

Supplemental Figure SC8: Confocal image of a whitefly egg attached to a transgenic Ly62 plant. The fluorescence of the vein is guided through the egg giving it a false perceived fluorescence. Fluorescent image with 550-570nm excitation, 570-640nm emission.

## Slide 9
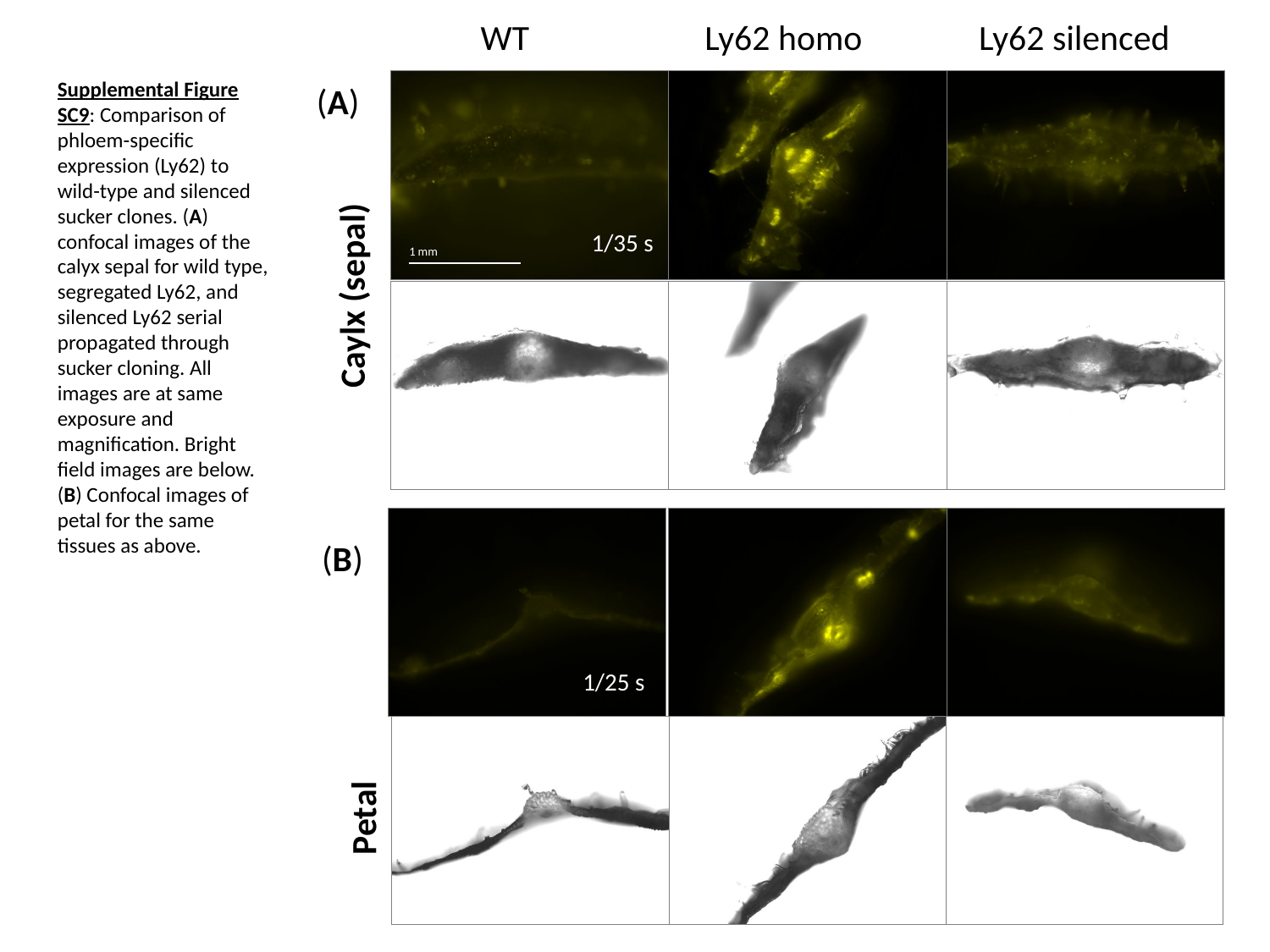

WT
Ly62 homo
Ly62 silenced
Supplemental Figure SC9: Comparison of phloem-specific expression (Ly62) to wild-type and silenced sucker clones. (A) confocal images of the calyx sepal for wild type, segregated Ly62, and silenced Ly62 serial propagated through sucker cloning. All images are at same exposure and magnification. Bright field images are below. (B) Confocal images of petal for the same tissues as above.
(A)
1/35 s
1 mm
Caylx (sepal)
(B)
1/25 s
Petal

## Slide 10
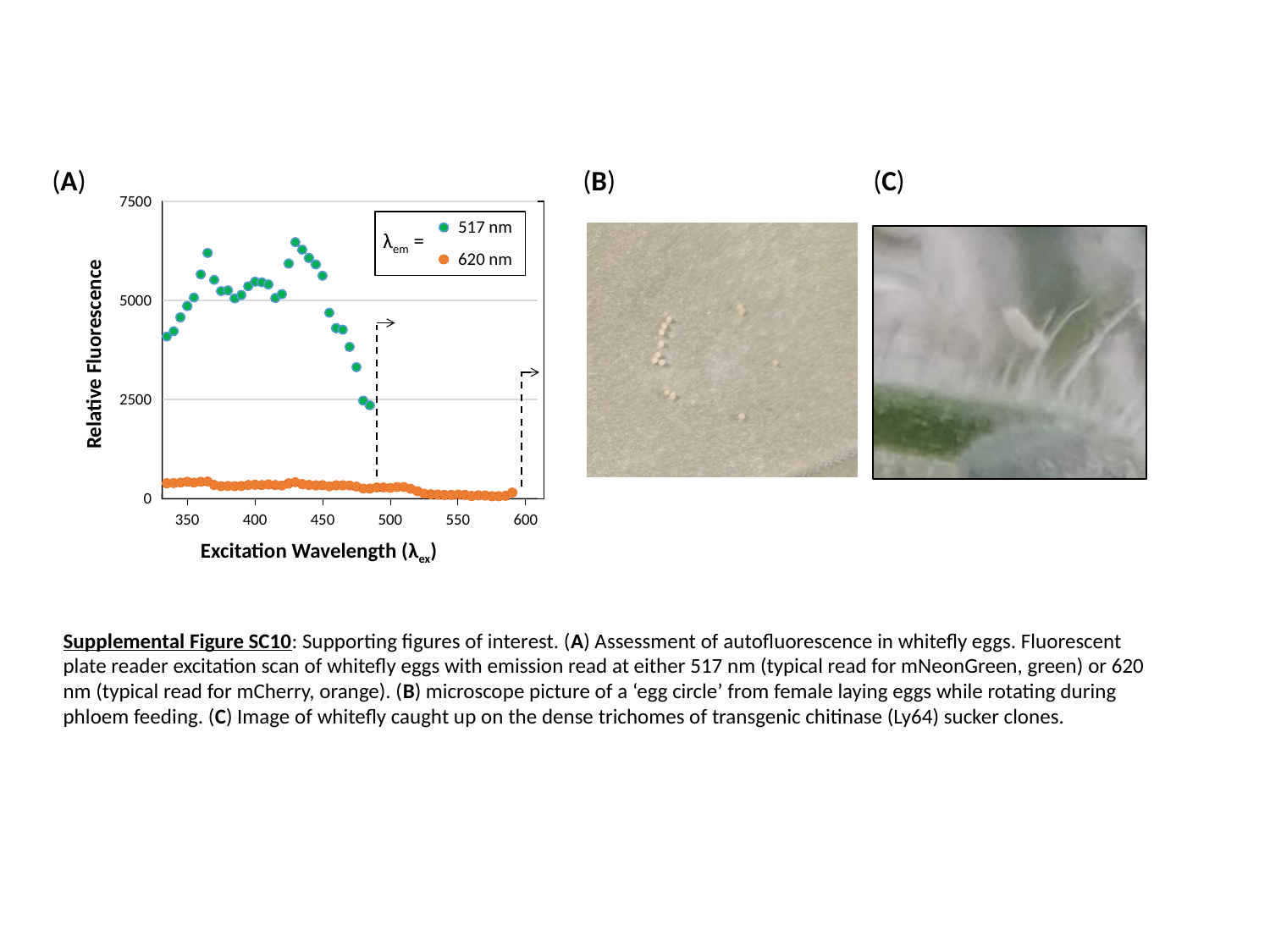

(A)
(B)
(C)
7500
517 nm
λem =
620 nm
5000
Relative Fluorescence
2500
0
350
400
450
500
550
600
Excitation Wavelength (λex)
Supplemental Figure SC10: Supporting figures of interest. (A) Assessment of autofluorescence in whitefly eggs. Fluorescent plate reader excitation scan of whitefly eggs with emission read at either 517 nm (typical read for mNeonGreen, green) or 620 nm (typical read for mCherry, orange). (B) microscope picture of a ‘egg circle’ from female laying eggs while rotating during phloem feeding. (C) Image of whitefly caught up on the dense trichomes of transgenic chitinase (Ly64) sucker clones.

## Slide 11
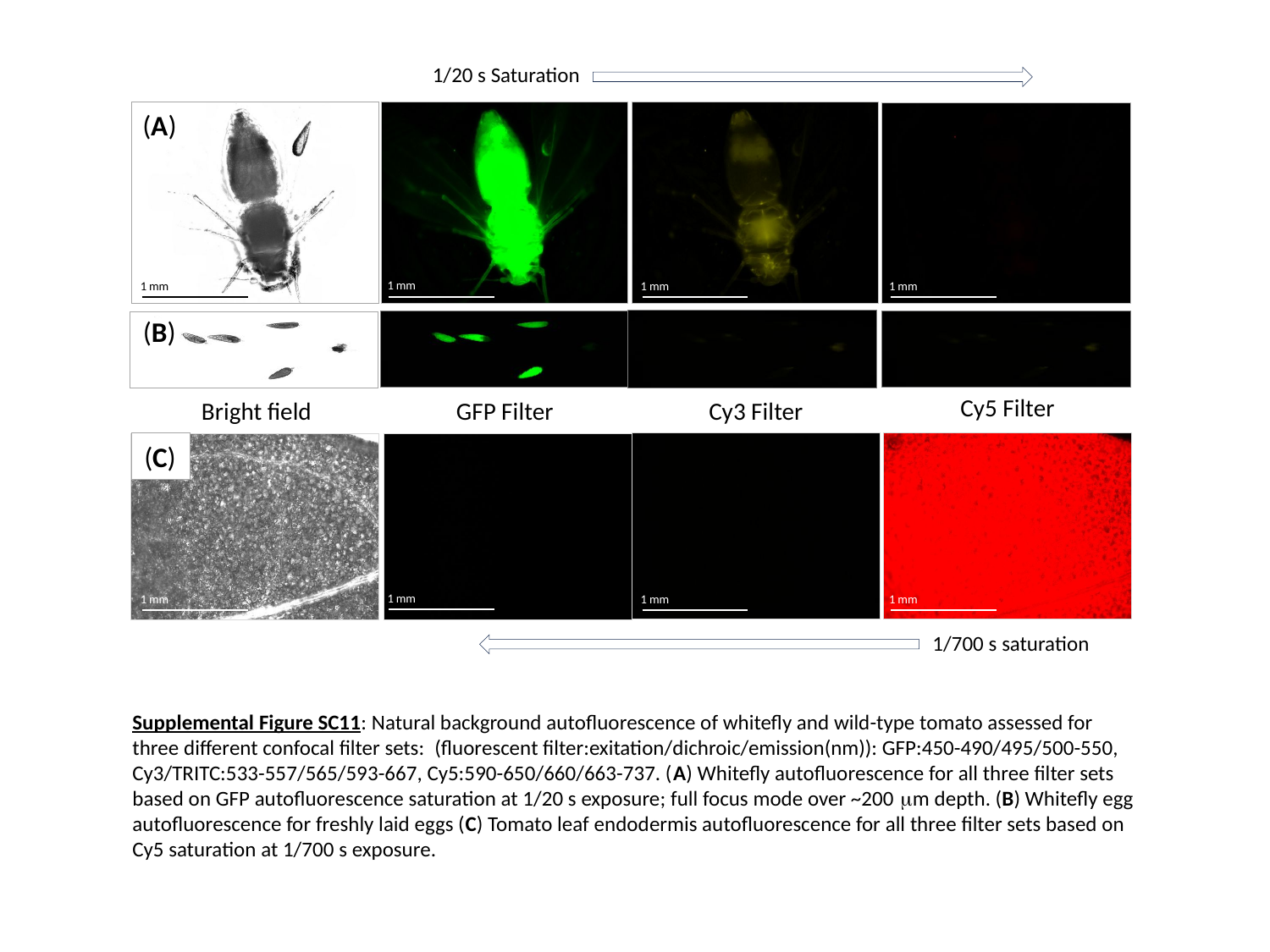

1/20 s Saturation
(A)
1 mm
1 mm
1 mm
1 mm
(B)
Cy5 Filter
Bright field
GFP Filter
Cy3 Filter
(C)
1 mm
1 mm
1 mm
1 mm
1/700 s saturation
Supplemental Figure SC11: Natural background autofluorescence of whitefly and wild-type tomato assessed for three different confocal filter sets:  (fluorescent filter:exitation/dichroic/emission(nm)): GFP:450-490/495/500-550, Cy3/TRITC:533-557/565/593-667, Cy5:590-650/660/663-737. (A) Whitefly autofluorescence for all three filter sets based on GFP autofluorescence saturation at 1/20 s exposure; full focus mode over ~200 mm depth. (B) Whitefly egg autofluorescence for freshly laid eggs (C) Tomato leaf endodermis autofluorescence for all three filter sets based on Cy5 saturation at 1/700 s exposure.
1 mm

## Slide 12
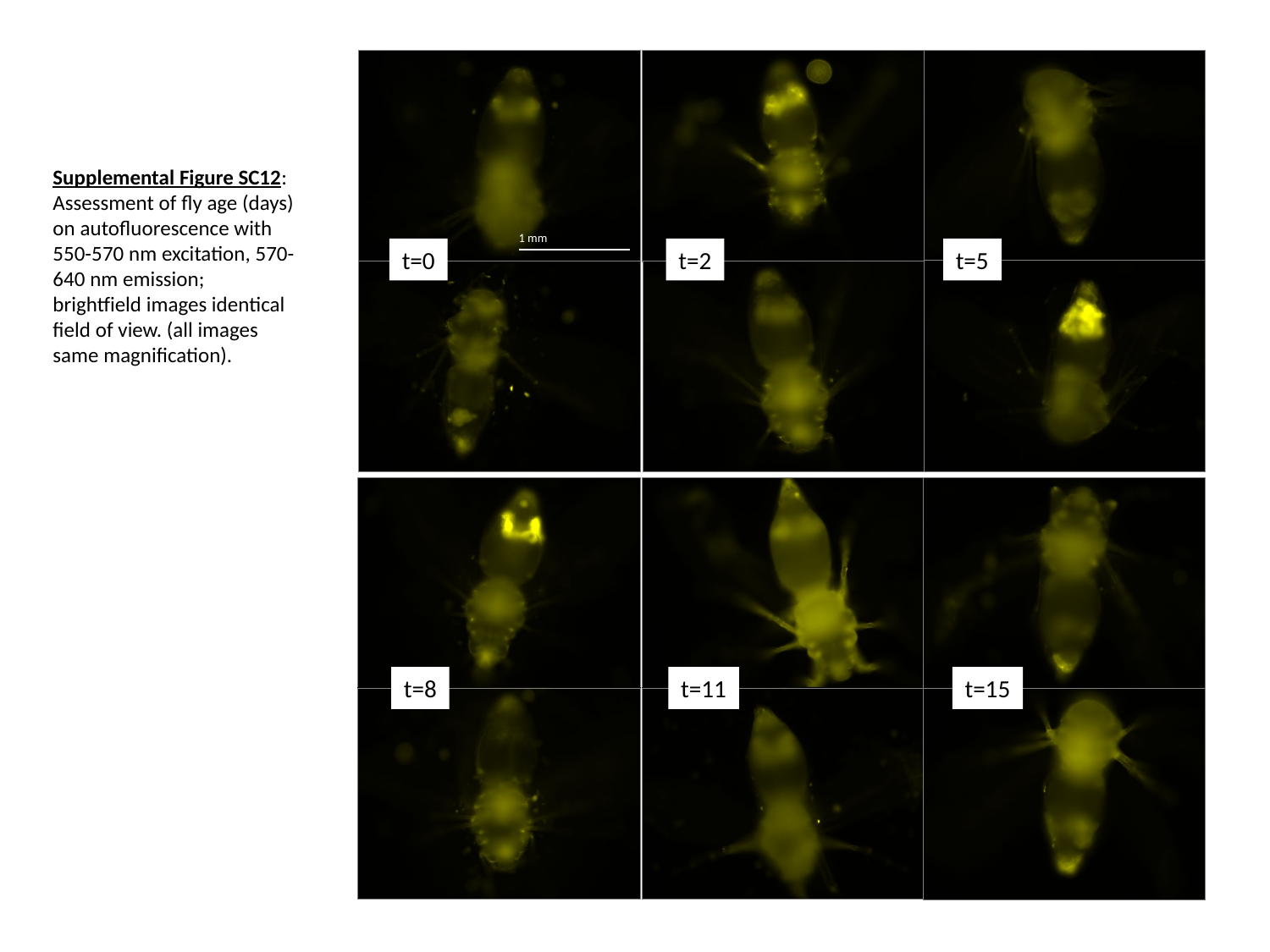

Supplemental Figure SC12: Assessment of fly age (days) on autofluorescence with 550-570 nm excitation, 570-640 nm emission; brightfield images identical field of view. (all images same magnification).
1 mm
t=2
t=5
t=0
t=8
t=11
t=15
