# Supplementary material for: Transgenic tomato strategies targeting whitefly eggs from apoplastic or ovary-directed proteins
Source: BMC Plant Biol. 2024 Dec 27;24:1262. doi: 10.1186/s12870-024-05852-5 (PMC11673810; doi:10.1186/s12870-024-05852-5)
Supplement: Supplementary file 4 — Supplementary Material 4: Supplemental File D: Examination for Whitefly Feeding on mCherry Transgenic Tomato [file 12870_2024_5852_MOESM4_ESM.docx]

**Supplemental File D – Examination for Whitefly Feeding on mCherry Transgenic Tomato**

Whiteflies feeding on Ly60 plants that were confirmed to be strongly expressing apoplastic mCherry did not display fluorescence that was elevated relative to control. In an attempt to see if a protein extract could provide better resolution, 10 whiteflies were macerated and subjected to fluorimetry in a 96-well plate format; there was still no difference.


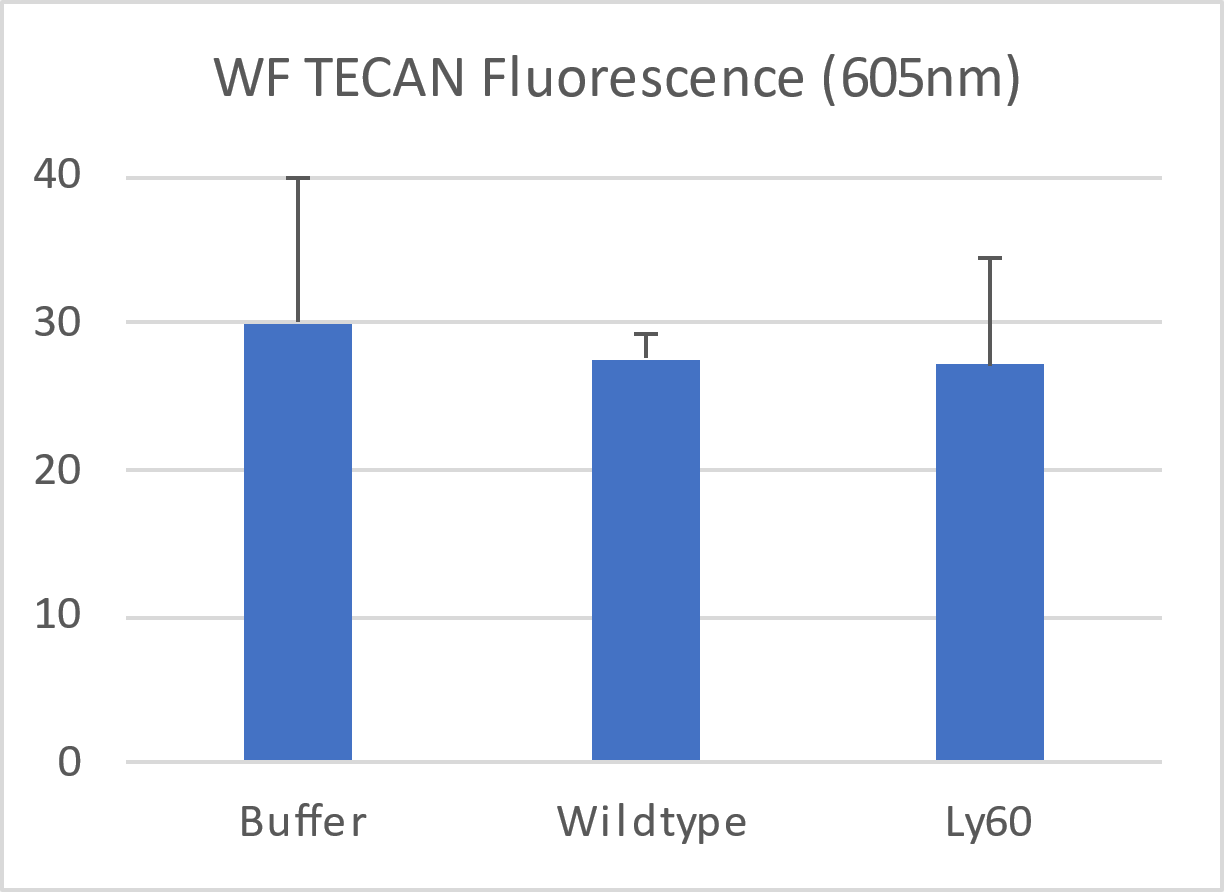


To avoid interference of the mCherry expressing transgenics, the whiteflies feeding on these plants were collected for microscopic examination (right, below). These were compared and did not display an autofluorescence relative to whiteflies feeding on wild-type tomato (center, below) or those removed from the plant to reduce interference (left, below).

| Fly fed on Ly62.1 | Fly fed on wild-type tomato | Fly fed on Ly62.1 detached from the plant for minimal interference |
| --- | --- | --- |
| 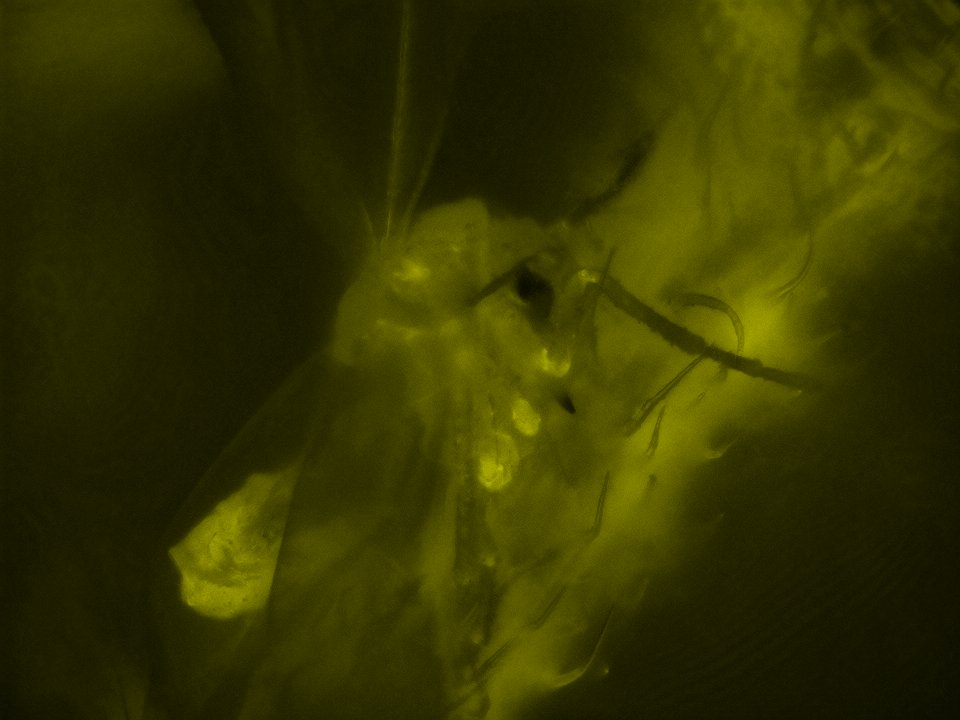 | 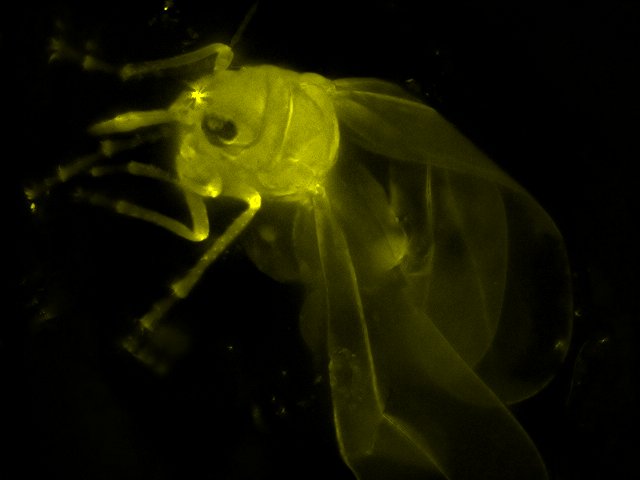 | 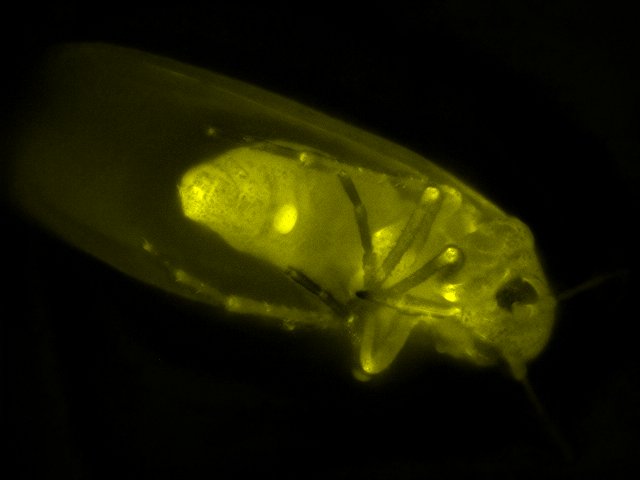 |

Additionally, the eggs themselves were looked at under similar conditions. Eggs that were laid on Ly62.1 (left, below) were compared to eggs that had been laid on cabbage by adults who had fed on wild-type tomato (center, below) and eggs that had been laid on cabbage by adults who had fed on Ly62.1 (right, below). No discernable difference was observed in overall fluorescence levels.

| Eggs laid on Ly62.1 | Eggs laid on cabbage by adults who had fed on WT tomato | Eggs laid on cabbage by adults who had fed on Ly62.1 |
| --- | --- | --- |
| 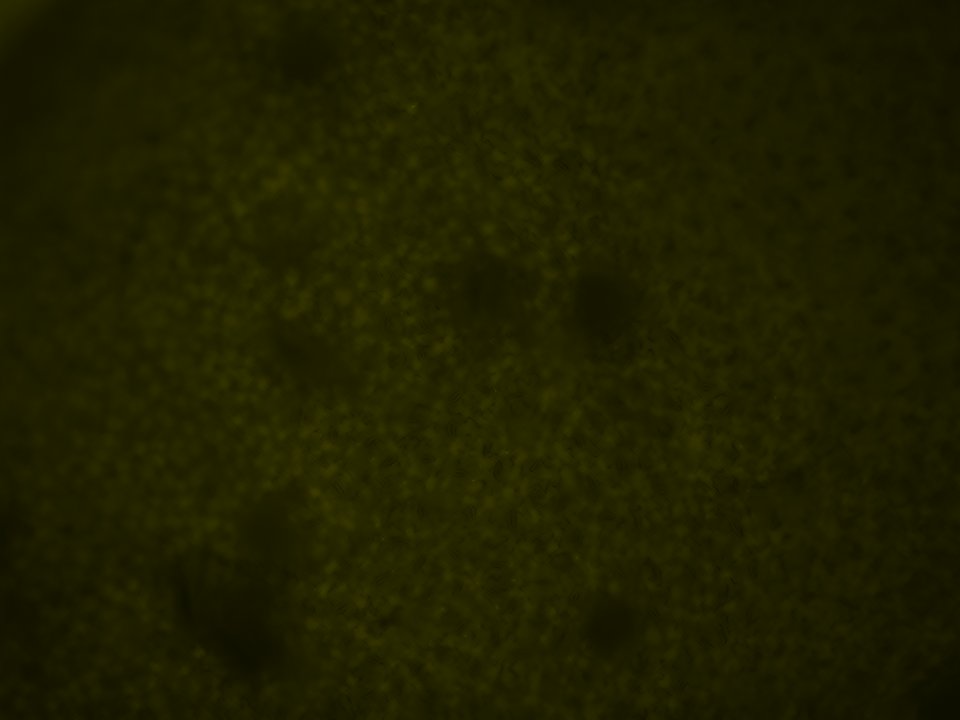 | 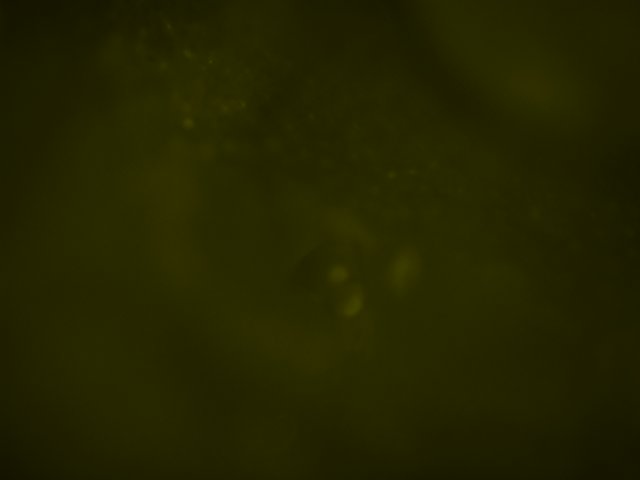 | 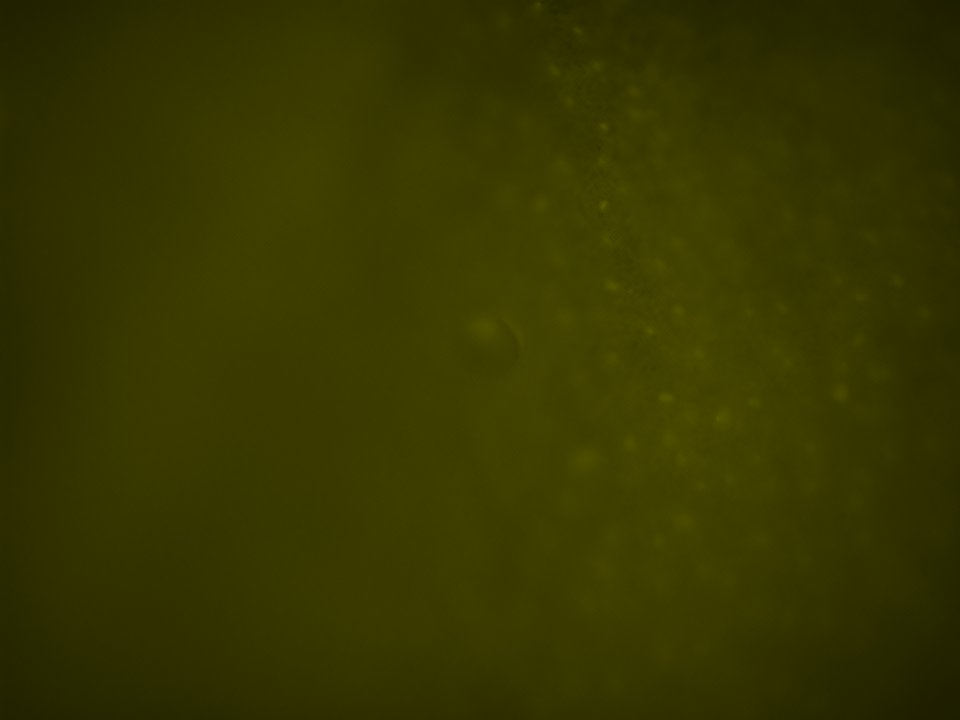 |

Noting that feeding whiteflies did display mCherry fluorescence, the conclusion of the aggregate of these results is that either that functional mCherry is not transported into the egg in the ovary of feeding female, or the levels are too low to be measured by our microscopy and spectroscopy methods.
