# Supplementary material for: Transgenic tomato strategies targeting whitefly eggs from apoplastic or ovary-directed proteins
Source: BMC Plant Biol. 2024 Dec 27;24:1262. doi: 10.1186/s12870-024-05852-5 (PMC11673810; doi:10.1186/s12870-024-05852-5)
Supplement: Supplementary file 5 — Supplementary Material 5: Supplemental File E: Tomato Transgenic for Tma12 Chitinase [file 12870_2024_5852_MOESM5_ESM.docx]

**Supplemental File E – Tomato Transgenic for Tma12 Chitinase**

The whitefly proliferation rate on tomato was observed to vary wildly both within a given experiment as well as between different experiments. For instance, in experiment SC1 (Sucker-clone 1) as shown graphically below, 10 sucker clones were compared with a standard inoculum of 30 ± 1 adult whiteflies. Nine of these clones were from nine separate segregations of either Ly61 or Ly64 (red bars) with the remaining clone being from a wild-type *Florida lanai* as a negative control (green bar). The average resultant number of whiteflies amongst the transgenic chitinase tomato plants was 633 ± 405 whiteflies. This large variability is likely due to several factors: (1) sucker clone morphology is prone to hunched and bent stems as well as severely curled leaves, likely inhibiting access for whiteflies to proliferate, (2) a small inoculum of 30 whiteflies may be effected by the unknown ratio of male to female adults, and (3) unknown transgene copy number across the different segregants of the original T0 initial transformant as well as issues of gene silencing observed for the mCherry sucker clones. Notably, the wild-type ‘Florida Lanai’ plant resulted in a net total of 279 whiteflies which is less than the average of the transgenic tomatoes.

| **First cage study (SCR1)** comparing 9 different sucker clones of transgenic chitinase (Ly61 & Ly64) plants to a wild-type control. |
| --- |
| 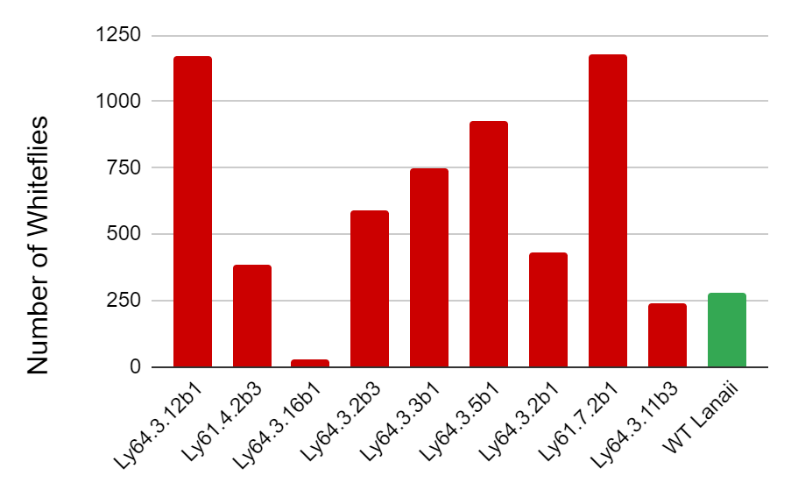 |

We point out that for cabbage plants executed in this manner as a quantitative model of whitefly proliferation (https://doi.org/10.1111/jen.12995), the numbers of proliferated whitefly in this time period would be about 5000, suggestive of considerable innate resistance mechanisms against whitefly in tomato. Beyond trichomes, are the extensive repertoire of tomato chitinases (see Supplemental File F).

Two subsequent sucker clone studies (SC2 and SC3) attempted to address this high variability with replicates of a consistent transgenic line that had initially performed well in a fungal assay (see main text) but did not see improvement in the final harvest consistency.

One perceived issue was related to the differences in age of the sucker clones and as a consequence, initial surface area. To ascertain if there is a higher expression level of native chitinases and other insecticidal proteins at different ages of tomato plants, we tested three different initial ages of tomato plants in cage studies. To distinguish initial tomato host surface area from plant age at the time of whitefly addition, we added more of the younger plants to approximate the same initial surface area. The results showed no statistical difference between the age of the plant and the number of whiteflies produced based on a consistent initial inoculation quantity.

Another hypothesis that was tested was the observation of a sucker clone being morphologically different – in trichome density in particular - from a “fresh” plant that has been started from seed. Another cage study (Seed1) was conducted with three replicates of wild-type ‘Florida Lanai’ and three replicates of a segregant of Ly64. All plants were started from seed and presence of the chitinase transgene was confirmed via PCR in the Ly64 plants prior to experimentation. The results were highly variable and showed no statistical difference between transgenic and wild-type.

| **Final Cage Study (Seed1)** comparing three replicates of wild-type ‘Florida Lanai’ (WT Lanai, left green bars) with three replicates of one transgenic line of Ly64’s (right red bars). (all started from seed) |
| --- |
|  |

Other potential problematic issues of this chitinase-tomato insecticidal pair are discussed in the main text of the manuscript. We have notably developed an axenic whitefly colony methodology that includes methods of aseptic feeding of whitefly (N. Thompson et al., 2024, Scientific Reports, [https://doi:10.1038/s41598-024-73583-6](about:blank)). This would now provide the opportunity of pre-screening the potential for reducing fecundity via feeding studies as a valuable way of demonstrating insecticidal activity, prior to generating the transgenics.
