# Supplementary material for: Transgenic tomato strategies targeting whitefly eggs from apoplastic or ovary-directed proteins
Source: BMC Plant Biol. 2024 Dec 27;24:1262. doi: 10.1186/s12870-024-05852-5 (PMC11673810; doi:10.1186/s12870-024-05852-5)
Supplement: Supplementary file 6 — Supplementary Material 6: Supplemental File F: Native Chitinase Genes in Tomato [file 12870_2024_5852_MOESM6_ESM.docx]

**Supplemental F – Native Chitinase Genes in Tomato**

This supplemental file contains the Genbank accession numbers for gene DNA sequences that are annotated as related to chitinase in the tomato genome. A phylogenetic tree was generated based on the protein sequence using MUSCLE, where the fern (*Tectaria macrodonta*) Tma12 transgene is quite distinct from those of tomato.


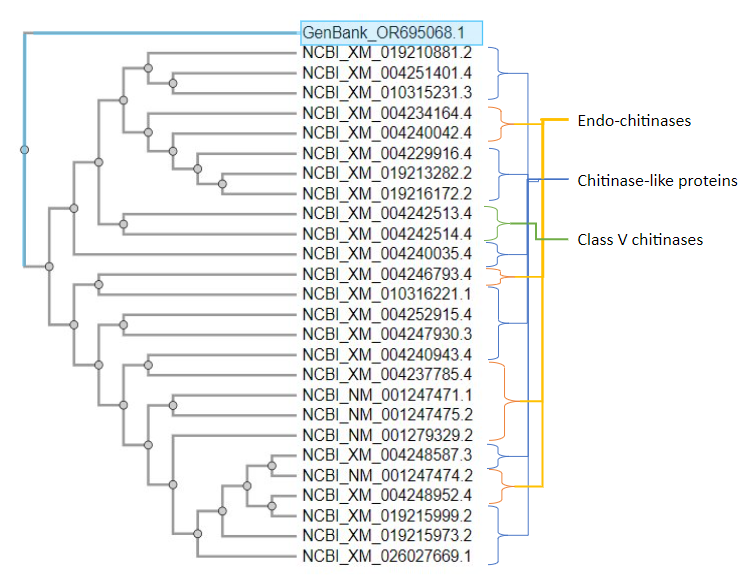


Figure F1: Chitinases from *Solanum lycopersicum*

The vast majority of chitinases expressed in tomato are **endo-chitinases** (which split chitin at internal sites) rather than exo-chitinases. Additionally, there are a fair number of **chitinase-like proteins** – proteins that bind to chitin but do not catalyze it. **Class V chitinases** are not well characterized, but two of the tomato chitinases fall in this class. Each sequence in the figure is referenced by its NCBI accession number. The only exception is the GenBank reference which is the *Tma1* fern chitinase that is used in this paper. Unsurprisingly, the fern sequence does not show significant similarity to analogous tomato chitinases.

Tomato chitinase genes

A search was performed on Genbank for chitinase genes from the genus *Solanum lycopsersicum* with the following results:

>Sequence 1 –[GENBANK](https://www.ncbi.nlm.nih.gov/nucleotide/XM_004242930.4?report=genbank&log$=nuclalign&blast_rank=1&RID=DJJGJ9VA013&from=1568&to=2725):NCBI Reference Sequence: XM_004242930.4

Solanum lycopersicum extensin-like (LOC101243800), mRNA

>Sequence 2 –[GENBANK](https://www.ncbi.nlm.nih.gov/nucleotide/XM_004240943.4?report=genbank&log$=nuclalign&blast_rank=1&RID=DJJDV6FW016&from=59&to=928): NCBI Reference Sequence: XM_004240943.4

Solanum lycopersicum endochitinase PR4-like (LOC101243897), mRNA

>Sequence 3 –[GENBANK](https://www.ncbi.nlm.nih.gov/nucleotide/XM_004229916.4?report=genbank&log$=nuclalign&blast_rank=1&RID=DJJ9R92M013&from=82&to=990): NCBI Reference Sequence: XM_004229916.4

Solanum lycopersicum endochitinase-like (LOC101246963), Mnra

>Sequence 4 –[GENBANK](https://www.ncbi.nlm.nih.gov/nucleotide/XM_004248952.4?report=genbank&log$=nuclalign&blast_rank=1&RID=DJJ570FW016&from=19&to=987): NCBI Reference Sequence: XM_004248952.4

Solanum lycopersicum endochitinase (LOC101251136), mRNA

>Sequence 5 –[GENBANK](https://www.ncbi.nlm.nih.gov/nucleotide/XM_004240035.4?report=genbank&log$=nuclalign&blast_rank=1&RID=DJJ2EW7M013&from=78&to=1409): NCBI Reference Sequence: XM_004240035.4

Solanum lycopersicum chitinase domain-containing protein 1 (LOC101251382), transcript variant X1, mRNA

>Sequence 6 –[GENBANK](https://www.ncbi.nlm.nih.gov/nucleotide/XM_004252915.4?report=genbank&log$=nuclalign&blast_rank=1&RID=DJJ0C8G7013&from=195&to=1181):NCBI Reference Sequence: XM_004252915.4

Solanum lycopersicum chitinase-like protein 1 (LOC101252859), mRNA

>Sequence 7 –[GENBANK](https://www.ncbi.nlm.nih.gov/nucleotide/XM_004240042.4?report=genbank&log$=nuclalign&blast_rank=1&RID=DJHXVSVT01N&from=51&to=929):NCBI Reference Sequence: XM_004240042.4

Solanum lycopersicum acidic endochitinase (LOC101253788), mRNA

>Sequence 8 –[GENBANK](https://www.ncbi.nlm.nih.gov/nucleotide/XM_004247930.3?report=genbank&log$=nuclalign&blast_rank=1&RID=DJHVR1P401N&from=194&to=1153): NCBI Reference Sequence: XM_004247930.3

Solanum lycopersicum chitinase-like protein 1 (LOC101254426), mRNA

>Sequence 9 –[GENBANK](https://www.ncbi.nlm.nih.gov/nucleotide/XM_004237785.4?report=genbank&log$=nuclalign&blast_rank=1&RID=DJHTP2M801N&from=35&to=865): NCBI Reference Sequence: XM_004237785.4

Solanum lycopersicum endochitinase EP3 (LOC101256086), mRNA

>Sequence 10 –[GENBANK](https://www.ncbi.nlm.nih.gov/nucleotide/XM_004242513.4?report=genbank&log$=nuclalign&blast_rank=1&RID=DJHRUE1N01N&from=61&to=1176):  NCBI Reference Sequence: XM_004242513.4

Solanum lycopersicum class V chitinase (LOC101257483), mRNA

>Sequence 11 –[GENBANK](https://www.ncbi.nlm.nih.gov/nucleotide/XM_004242514.4?report=genbank&log$=nuclalign&blast_rank=1&RID=DJHNTYMN013&from=8&to=1138): NCBI Reference Sequence: XM_004242514.4

Solanum lycopersicum class V chitinase (LOC101257783), mRNA

>Sequence 12 –[GENBANK](https://www.ncbi.nlm.nih.gov/nucleotide/XM_019215973.2?report=genbank&log$=nuclalign&blast_rank=1&RID=DJHKJT8M013&from=1&to=519): NCBI Reference Sequence: XM_019215973.2

Solanum lycopersicum endochitinase A-like (LOC101258911)

>Sequence 13 –[GENBANK](https://www.ncbi.nlm.nih.gov/nucleotide/XM_026027669.1?report=genbank&log$=nuclalign&blast_rank=1&RID=DJHGDGVM013&from=189&to=1134): NCBI Reference Sequence: XM_026027669.1;

Solanum lycopersicum endochitinase-like (LOC101259497), mRNA

>Sequence 14 –[GENBANK](https://www.ncbi.nlm.nih.gov/nucleotide/XM_004234164.4?report=genbank&log$=nuclalign&blast_rank=1&RID=DJHCJHAF01N&from=224&to=1108): NCBI Reference Sequence: XM_004234164.4;

Solanum lycopersicum acidic endochitinase (LOC101262490), mRNA

>Sequence 15 –[GENBANK](https://www.ncbi.nlm.nih.gov/nucleotide/XM_004248030.4?report=genbank&log$=nuclalign&blast_rank=1&RID=DJHA8U6001N&from=157&to=1617): NCBI Reference Sequence: XM_004248030.4

Solanum lycopersicum rhodanese-like domain-containing protein 4, chloroplastic (LOC101262961), mRNA

>Sequence 16 –[GENBANK](https://www.ncbi.nlm.nih.gov/nucleotide/XM_019210881.2?report=genbank&log$=nuclalign&blast_rank=1&RID=DJH789S801N&from=1753&to=2562): NCBI Reference Sequence: XM_019210881.2;

Solanum lycopersicum chitinase 2-like (LOC101264585), mRNA

>Sequence 17 –[GENBANK](https://www.ncbi.nlm.nih.gov/nucleotide/XM_004251401.4?report=genbank&log$=nuclalign&blast_rank=1&RID=DJH2F1RT013&from=755&to=1783): NCBI Reference sequence: XM_004251401.4

Solanum lycopersicum chitinase 2-like (LOC101265509), mRNA

>Sequence 18 –[GENBANK](https://www.ncbi.nlm.nih.gov/nucleotide/XM_010315231.3?report=genbank&log$=nuclalign&blast_rank=1&RID=DJH0CF6C013&from=215&to=1135): NCBI Reference Sequence: XM_010315231.3;

Solanum lycopersicum chitinase 2-like (LOC101265800), mRNA

>Sequence 19 –[GENBANK](https://www.ncbi.nlm.nih.gov/nucleotide/XM_019215999.2?report=genbank&log$=nuclalign&blast_rank=1&RID=DJGY9FM6013&from=151&to=546): NCBI Reference Sequence: XM_019215999.2;

Solanum lycopersicum endochitinase A-like (LOC101265876), mRNA

>Sequence 20 –[GENBANK](https://www.ncbi.nlm.nih.gov/nucleotide/XM_019213282.2?report=genbank&log$=nuclalign&blast_rank=1&RID=DJGVEN1P013&from=152&to=1048): NCBI Reference Sequence: XM_019213282.2;

Solanum lycopersicum acidic endochitinase-like (LOC101266570), mRNA

>Sequence 21 –[GENBANK](https://www.ncbi.nlm.nih.gov/nucleotide/XM_004248587.3?report=genbank&log$=nuclalign&blast_rank=1&RID=DJGS97FS013&from=83&to=1075): NCBI Reference Sequence: XM_004248587.3;

Solanum lycopersicum endochitinase 3-like (LOC101266770), mRNA

>Sequence 22 –[GENBANK](https://www.ncbi.nlm.nih.gov/nucleotide/XM_004246793.4?report=genbank&log$=nuclalign&blast_rank=1&RID=DJGNY0CJ013&from=267&to=1874): NCBI Reference Sequence: XM_004246793.4;

Solanum lycopersicum endochitinase A (LOC101267157), mRNA

>Sequence 23 –[GENBANK](https://www.ncbi.nlm.nih.gov/nucleotide/XM_010316221.1?report=genbank&log$=nuclalign&blast_rank=1&RID=DJGH4BR8013&from=1&to=1335): NCBI Reference Sequence: XM_010316221.1;

Solanum lycopersicum endochitinase A-like (LOC104645143), mRNA

>Sequence 24 –[GENBANK](https://www.ncbi.nlm.nih.gov/nucleotide/XR_743355.3?report=genbank&log$=nuclalign&blast_rank=1&RID=DJG8P3XJ01N&from=133&to=1461): NCBI Reference Sequence: XR_743355.3

Solanum lycopersicum rhodanese-like domain containing protein 4, chloroplastic (LOC104649438), transcript X2, misc-RNA

>Sequence 25 –[GENBANK](https://www.ncbi.nlm.nih.gov/nucleotide/XM_019216172.2?report=genbank&log$=nuclalign&blast_rank=1&RID=DJG2FAP101N&from=915&to=1811): NCBI Reference Sequence: XM_019216172.2;

Solanum lycopersicum acidic endochitinase-like

>Sequence 26 –[GENBANK](https://www.ncbi.nlm.nih.gov/nucleotide/NM_001279329.2?report=genbank&log$=nuclalign&blast_rank=1&RID=DJFJ9BZ8013&from=14&to=805): NCBI Reference Sequence: NM_001279329.2;

Solanum lycopersicum chitinase (CHI14)

>Sequence 27 –[GENBANK](https://www.ncbi.nlm.nih.gov/nucleotide/NM_001247471.1?report=genbank&log$=nuclalign&blast_rank=2&RID=DJF97GKG013&from=17&to=760): NCBI Reference Sequence: NM_001247471.1;

Solanum lycopersicum chitinase (CHI17)

>Sequence 28 –[GENBANK](https://www.ncbi.nlm.nih.gov/nucleotide/NM_001247474.2?report=genbank&log$=nuclalign&blast_rank=1&RID=DJF64DPE016&from=33&to=1001): NCBI Reference Sequence: NM_001247474.2;

Solanum lycopersicum chitinase (CHI9)

>Sequence 29 –[GENBANK](https://www.ncbi.nlm.nih.gov/nucleotide/NM_001247475.2?report=genbank&log$=nuclalign&blast_rank=1&RID=DJEZHHDB013): NCBI Reference Sequence: NM_001247475.2;

Solanum lycopersicum chitinase (CHI3)
