# Supplementary material for: Transgenic tomato strategies targeting whitefly eggs from apoplastic or ovary-directed proteins
Source: BMC Plant Biol. 2024 Dec 27;24:1262. doi: 10.1186/s12870-024-05852-5 (PMC11673810; doi:10.1186/s12870-024-05852-5)
Supplement: Supplementary file 7 — Supplementary Material 7: Supplemental File G: Additional Cloning Information / Constructs [file 12870_2024_5852_MOESM7_ESM.docx]

**Supplemental G – Additional Cloning Information / Constructs**

This supplementary file contains DNA sequences utilized in creating the ovary-targeting constructs using overlap-extension PCR. This includes elements of the protein transduction domain (PTD) as well as the synthetic vitellogenin domain (Syn / SynVG).

**Table G1** - Primer list for ovary-targeting mCherry and Tma12 chitinase

| Primer | Sequence | Description |
| --- | --- | --- |
| nDPTD SacII Fwd | 5’GGGGCCGCGGATGAGGCAAATCAAGATTTGGTTTCAA 3’ | End primer (forward) for cloning PTD:SynVG :mCherry in pLSU2 |
| nmCherry MluI Rev | 5’GGGGACGCGTTTACTCGTCCATGCCGCCGGTGCTGTG 3’ | End primer (Reverse) for cloning PTD:SynVG :mCherry in pLSU2 |
| SynTag F1 SacII Fwd | 5’TTTACCGGTGTCGACCCGCGGATGTGGGAACTGAATATCATTAAGG 3’ | End primer (Forward) for cloning SynVG: mCherry: PTD in pLSU2 |
| DPTD R1 MluI Rev | 5’GGGGCTCGAGACGCGTTTTTTTCCACTTCATCCTTCGGTTTTGAAACCAAATCTTGATTTGCCT 3’ | End primer (Reverse) for cloning SynVG: mCherry: PTD in pLSU2 |
| mCherryDPTD Olap Fwd | 5’ CGAGCTGTACAAGTAAAGGCAAATCAAGATTTGG 3’ | Forward primer overlap fusion PCR for PTD and mCherry gene |
| mCherryDPTD Olap Rev | 5’ CCAAATCTTGATTTGCCTTTACTTGTACAGCTCG 3’ | Reverse primer overlap fusion PCR for PTD and mCherry gene |
| SynmCherry Olap Fwd | 5’TAATTACAGTAAATGCATGGCCATCATCAAGGAGTTCATG 3’ | Forward primer overlap fusion PCR for Synthetic Vg and mCherry gene |
| SynmCherry Olap Rev | 5’CATGAACTCCTTGATGATGGCCATGCATTTACTGTAATTA 3’ | Reverse primer overlap fusion PCR for Synthetic Vg and mCherry gene |
| nDPTDSynVg Fwd | 5’AGGCAAATCAAGATTTGGTTTCAAAACCGAAGGATGAAGTGGAAAAAATGGGAACTGAATATCAT 3’ | Forward Primer overlap fusion for PTD and Synthetic Vg |
| nDPTDSynVg Rev | 5’ATGATATTCAGTTCCCATTTTTTCCACTTCATCCTTCGGTTTTGAAACCAAATCTTGATTTGCCT 3’ | Reverse Primer overlap fusion for PTD and Synthetic Vg |
| SynTag-Chi-Olap_F | 5’ TAATTACAGTAAATGCATGGGAAGAAGTTGG 3’ | Forward primer overlap fusion PCR for Synthetic Vg and Tma12 gene |
| SynTag-Chi-Olap_R | 5’CCAACTTCTTCCCATGCATTTACTGTAATTA 3’ | Reverse primer overlap fusion PCR for Synthetic Vg and mCherry gene |
| Chit MluI Rev | 5’TTTTCTCGAGACGCGTCCCGGGCTAAGTGGTGCTATGTAAACTG 3’ | End primer (Reverse) for cloning PTD:SynVG :Chitinase in pLSU2 |
| Chit-PTD Olap Fwd | 5’CATAGCACCACTTAGAGGCAAATCAAGATTTGGTTTCAA 3’ | Forward primer overlap fusion PCR for PTD and Chitinase gene |
| Chit-PTD Olap Rev | 5’TTGAAACCAAATCTTGATTTGCCTCTAAGTGGTGCTATG 3’ | Reverse primer overlap fusion PCR for PTD and Chitinase gene |

Top of Form

Sequences:

SynVG

TGGGAACTGAATATCATTAAGGCTGTGGTGTCACAAATACAACAGAATCTGAAGAAAAGTTCCTATAAAACTATGGAGGACAGCGTCACTGGAGAGTGCGAGACATTGTACGATGTCAGCCAATTTATTGATATAGTCAAAACAACTAATTACAGTAAATGC

mCherry Gene

ATGGCCATCATCAAGGAGTTCATGCGGTTCAAGGTGCACATGGAAGGCAGCGTGAACGGCCACGAGTTCGAGATCGAGGGCGAGGGCGAAGGCAGGCCCTACGAGGGCACCCAGACCGCCAAGCTGAAGGTGACCAAGGGCGGACCTCTGCCCTTCGCCTGGGACATCCTGAGCCCCCAGTTCATGTACGGCAGCAAGGCCTACGTGAAGCACCCCGCCGACATCCCCGACTACCTGAAGCTGTCCTTCCCCGAGGGATTCAAATGGGAGCGGGTGATGAACTTCGAGGACGGCGGCGTGGTGACCGTGACCCAGGACAGCAGCCTGCAGGACGGCGAGTTCATCTACAAGGTGAAACTGCGGGGCACCAACTTCCCCAGCGACGGCCCCGTGATGCAGAAAAAGACCATGGGCTGGGAGGCCAGCAGCGAGCGGATGTACCCCGAGGATGGCGCCCTGAAGGGCGAGATCAAGCAGCGGCTGAAGCTGAAGGATGGCGGCCACTACGACGCCGAGGTGAAAACCACCTACAAGGCCAAGAAACCCGTGCAGCTGCCTGGCGCCTACAACGTGAACATCAAGCTGGACATCACCAGCCACAACGAGGACTACACCATCGTGGAGCAGTACGAGCGGGCCGAGGGCAGACACAGCACCGGCGGCATGGACGAGTAA

Tma12 Chitinase Gene (Tomato Plant Codon Optimized):

ATGGGAAGAAGTTGGGGAGTCGTCGCCGTCATGGTGCTCTGCGCTAGTGGACTACTAGGCATCGTGAGGGGGCATGGTTCAATGGAGGACCCAATTAGTAGAGTCTACAGGTGTAGATTAGAGAATCCTGAGAGGCCAACATCACCCGCTTGTCAGGCAGCTGTAGCACTAAGTGGTACCCAGGCTTTTTACGATTGGAACGAGGTCAACATTCCCAACGCAGCTGGAAGACATAGAGAGCTAATTCCTGATGGCCAGCTCTGTTCAGCTGGCCGATTCAAATTCAGGGGATTAGACTTGGCAAGAAGTGATTGGATCGCTACTCCTCTCCCATCAGGTGCCTCTAGCTTTCCCTTCCGTTACATAGCTACAGCCGCTCACCTCGGCTTCTTCGAGTTCTACGTCACAAGGGAGGGTTATCAGCCAACTGTACCCTTAAAATGGGCTGACTTAGAAGAACTGCCATTCATAAACGTGACCAACCCACCTTTGGTAAGTGGGTCCTACCAAATCACTGGGACAACTCCTAGTGGTAAGTCAGGCAGTCATTTGATCTACGTAATATGGCAGCGTACAGACAGTCCCGAAGCTTTCTATTCATGCTCCGATGTCTATTTTACAGACGCCCTCAGTTTACATAGCACCACTTAG

CoYMV promoter

ATCGATTTCTTAGGGGCTTCTCTCGGATGTACAAAAATCAAGCTTCAGCCCCACATCATTTCAAAAATATGTGACTTCTCAGATGAAAAACTAGCAACACCTGAAGGTATGAGAAGCTGGTTGGGTATCCTCTCATATGCTAGAAATTATATTCAGGATATCGGCAAATTGGTGCAACCACTCAGACAAAAGATGGCACCAACAGGAGACAAGAGAATGAATCCAGAAACATGGAAGATGGTAAGACAGATAAAAGAAAAGGTGAAAAATCTCCCTGATCTTCAGTTACCACCTAAAGATTCATTCATCATAATAGAGACGGATGGTTGTATGACTGGCTGGGGAGCCGTCTGCAAATGGAAAATGTCAAAGCATGATCCAAGAAGCACCGAAAGAATTTGTGCCTATGCTAGTGGATCATTCAATCCAATAAAATCAACCATCGATGCAGAGATTCAGGCGGCAATCCATGGCCTGGATAAATTCAAAATTTATTATCTTGATAAAAAGGAGCTCATAATTCGCTCAGACTGTGAAGCAATTATCAAATTTTACAACAAGACGAACGAAAATAAGCCGTCTAGAGTTAGATGGTTAACATTTTCAGATTTCTTAACAGGTCTTGGAATCACAGTTACATTCGAGCACATAGATGGAAAGCATAATGGCTTAGCAGATGCTCTATCAAGAATGATAAATTTCATTGTGGAGAAAAATGATGAATCTCCATACAGGTTCACTTCATCAGTAGAGGACGCACTAAAGGTCTGCAATGATGATCACGGAAGAAATTTGATATCCGCCGTCATCAATGACATCATCACAGTACTGAGGAGATGAATACTTAGCCATGAAGTAGCGTGCGAATATTACCTATGCCTTTATTCGCAGCGTTAGTGGCACTGAAAGGCATAAAGTTTGTTCGTTCTTATCAAAAACGAATCTTATCTTTGTAACTTGGTTACCCGGTATGCCGGTTCCCAAGCTTTATTTCCTTATTTAAGCACTTGTGTAGTAGCTTAGAAAACCAACACAACAAGGATCC
